# Transposon sequencing reveals metabolic pathways essential for *Mycobacterium tuberculosis* infection

**DOI:** 10.1371/journal.ppat.1011663

**Published:** 2024-03-18

**Authors:** Alisha M. Block, Parker C. Wiegert, Sarah B. Namugenyi, Anna D. Tischler

**Affiliations:** Department of Microbiology and Immunology, University of Minnesota, Twin Cities Campus, Minneapolis, Minnesota, United States of America; Duke University, UNITED STATES

## Abstract

New drugs are needed to shorten and simplify treatment of tuberculosis caused by *Mycobacterium tuberculosis*. Metabolic pathways that *M*. *tuberculosis* requires for growth or survival during infection represent potential targets for anti-tubercular drug development. Genes and metabolic pathways essential for *M*. *tuberculosis* growth in standard laboratory culture conditions have been defined by genome-wide genetic screens. However, whether *M*. *tuberculosis* requires these essential genes during infection has not been comprehensively explored because mutant strains cannot be generated using standard methods. Here we show that *M*. *tuberculosis* requires the phenylalanine (Phe) and *de novo* purine and thiamine biosynthetic pathways for mammalian infection. We used a defined collection of *M*. *tuberculosis* transposon (Tn) mutants in essential genes, which we generated using a custom nutrient-rich medium, and transposon sequencing (Tn-seq) to identify multiple central metabolic pathways required for fitness in a mouse infection model. We confirmed by individual retesting and complementation that mutations in *pheA* (Phe biosynthesis) or *purF* (purine and thiamine biosynthesis) cause death of *M*. *tuberculosis* in the absence of nutrient supplementation *in vitro* and strong attenuation in infected mice. Our findings show that Tn-seq with defined Tn mutant pools can be used to identify *M*. *tuberculosis* genes required during mouse lung infection. Our results also demonstrate that *M*. *tuberculosis* requires Phe and purine/thiamine biosynthesis for survival in the host, implicating these metabolic pathways as prime targets for the development of new antibiotics to combat tuberculosis.

## Author summary

*Mycobacterium tuberculosis* causes more than 10 million new cases of active tuberculosis (TB) disease and ~1.3 million deaths worldwide each year. Individuals with active TB must take a combination of four antibiotics for a minimum of 6–9 months to cure the infection. New anti-tubercular drugs are needed to simplify TB treatment and combat drug resistance. Here, we describe a novel collection of *M*. *tuberculosis* mutants lacking metabolic pathways essential for growth in standard laboratory conditions. Using these mutants, a mouse infection model, and deep sequencing we identified those metabolic pathways that *M*. *tuberculosis* also requires during infection. We find that *M*. *tuberculosis* mutants that cannot synthesize purine nucleotides, riboflavin, or certain amino acids are unable to grow in mice. We also find that mutant strains which cannot synthesize purine nucleotides or the amino acid phenylalanine die rapidly in laboratory cultures without nutrient supplementation, suggesting that new drugs targeting these pathways would kill *M*. *tuberculosis*. Overall, our work reveals multiple metabolic pathways that *M*. *tuberculosis* requires during infection, which could be pursued as new targets for development of anti-tubercular drugs.

## Introduction

*Mycobacterium tuberculosis* caused more deaths worldwide in 2022 than any other bacterial infectious agent, in part due to the complexity of tuberculosis (TB) treatment, which requires 6–9 months of therapy with multiple antibiotics [1,2]. Multidrug-resistant *M*. *tuberculosis* (MDR-TB) accounts for ~4.2% of TB infections and is more challenging to treat, requiring use of less effective second-line agents [1,3]. Development of new anti-tubercular drugs will be critical to reduce the length of TB treatment and to combat antibiotic resistance. New drug regimens hold some promise for reducing TB treatment duration and for treating MDR-TB [4–6]. However, defining diverse new targets for TB drug development will be necessary to counteract antibiotic resistance, which has already been documented for all existing drugs and all new or repurposed TB drugs in the clinical pipeline [7]. Metabolic pathways that *M*. *tuberculosis* requires for *in vitro* growth and that are highly vulnerable to inhibition have been proposed as novel TB drug targets [8–11]. However, these metabolic pathways may not be required for *M*. *tuberculosis* growth during infection due to differences in nutrient availability in host tissues as compared to the *in vitro* growth medium. Defining the metabolic pathways that *M*. *tuberculosis* requires during mammalian infection will be essential to prioritize targets for TB drug development.

*M*. *tuberculosis* genes required during mammalian infection have been defined by genome-wide transposon (Tn) mutant screens in the mouse infection model [12–17]. However, these studies excluded analysis of genes that are essential for *M*. *tuberculosis* growth in standard culture conditions *in vitro* [11], as it is not possible to generate insertional mutations in these genes. In a few cases, auxotrophic *M*. *tuberculosis* mutant strains generated on medium supplemented with exogenous nutrients were used to demonstrate that specific central metabolic pathways are essential for growth in mice. These include pathways for synthesis of the amino acids Met, Arg, Pro and Trp [13,18–21]. Other metabolic pathways such as coenzyme A, biotin, and trehalose synthesis were shown to be essential during mouse infection using *M*. *tuberculosis* conditional gene expression strains [22–24]. Each of these pathways are being pursued as potential targets for TB drug development.

However, not all metabolic pathways essential for *M*. *tuberculosis* growth *in vitro* are also required during mammalian infection. For example, the *nadABC* genes, which encode enzymes for *de novo* NAD^+^ synthesis, are essential *in vitro* but dispensable in the host [25,26]. *M*. *tuberculosis* can scavenge the nicotinamide precursor from host tissue to synthesize NAD^+^ using an alternative salvage pathway [26]. This example highlights the importance of determining whether genes that *M*. *tuberculosis* requires for *in vitro* growth are also necessary during infection. Of the 625 genes annotated as essential for optimal growth of *M*. *tuberculosis* on standard Middlebrook medium [11], only a few have been directly tested to determine whether they are also required during infection because deletion mutant strains lacking these genes cannot be generated under standard culture conditions.

We previously developed an arrayed Tn mutant library using MtbYM rich medium, a custom medium that contains many additional carbon and nitrogen sources, amino acids, nucleotide precursors, co-factors and vitamins as compared to standard Middlebrook medium [27,28]. Based on genome-wide transposon-sequencing (Tn-seq) analysis, 118 genes annotated as essential for optimal growth on Middlebrook 7H10 are non-essential on MtbYM rich [27]. These include genes required for synthesis of amino acids (Arg, Trp, Ile, Val, Leu, Glu, Pro, Ser, Tyr, Gln, Phe, Met, Cys), vitamins and co-factors (riboflavin, folates, pantothenate, NAD), and purine nucleotides, as well as certain catabolic pathways, including glycolysis [27]. We reasoned that our unique collection of Tn mutant strains selected on MtbYM rich medium could be exploited to rapidly assess whether *M*. *tuberculosis* requires these conditionally essential metabolic pathways for growth and survival during infection.

Here, we show that *M*. *tuberculosis* requires the phenylalanine (Phe) biosynthesis and *de novo* purine nucleotide and thiamine biosynthesis pathways for growth in the mammalian host. We screened our collection of Tn mutants in conditionally essential genes in a mouse infection model using transposon sequencing (Tn-seq) and identified multiple central metabolic pathways that *M*. *tuberculosis* requires for fitness in host lung and spleen tissues. We confirm that mutation of *pheA* (Phe synthesis) or *purF* (purine/thiamine synthesis) causes death of *M*. *tuberculosis* in the absence of exogenous nutrient supplementation *in vitro* and in the lungs of aerosol-infected mice. Our results demonstrate roles for multiple metabolic pathways in *M*. *tuberculosis* fitness during infection and implicate the Phe and purine/thiamine biosynthetic pathways as novel targets for development of anti-tubercular drugs.

## Results

*M*. *tuberculosis* genes that are essential for growth in standard Middlebrook medium have been defined by saturating Tn mutagenesis screens [11]. We previously constructed an arrayed Tn mutant library in the *M*. *tuberculosis* Erdman strain using MtbYM rich medium, which contains many additional carbon sources, amino acids, metabolic intermediates, vitamins and co-factors that are not present in Middlebrook medium [27,28]. Our library contains 48 Tn mutants in genes annotated as “essential” in Middlebrook medium that are non-essential in MtbYM rich medium [11,27,28]. We refer to these genes as Middlebrook-Essential (M-ES) genes. To determine whether *M*. *tuberculosis* requires these M-ES genes for survival in the host, we isolated Tn mutants in M-ES genes from our arrayed library and screened them for fitness defects in mice using transposon sequencing (Tn-seq).

### Tn insertions in genes annotated as essential for growth on Middlebrook medium can be isolated on MtbYM rich medium

Our arrayed *M*. *tuberculosis* Erdman Tn library made on MtbYM rich medium contains a total of 105 unique Tn insertions in 48 M-ES genes (**S1 Table**). Of these, 20 mutants in 17 unique M-ES genes harbored the Tn insertion near the 5’ or 3’ end of the gene (within the first 5% or last 95% of the coding sequence). As these Tn insertions were unlikely to disrupt gene function, these mutants were excluded from this study (**Tab C in S1 Table**, Tn mutants excluded). We recovered 45 of the Tn mutants in M-ES genes from their mapped locations in our arrayed library on MtbYM rich agar and confirmed the Tn insertion site by PCR (**Tabs A and B in S1 Table**).

We previously determined that some mutants in our MtbYM rich Tn library have defects in production of the outer membrane lipid phthiocerol dimycocerosate (PDIM) due to secondary mutations, unlinked to the Tn [28]. The M-ES Tn mutants might be PDIM deficient due to similar secondary mutations. As *M*. *tuberculosis* requires PDIM for virulence [17], we confirmed that the M-ES Tn mutants were PDIM-proficient prior to screening for fitness defects in mice. We used PCR and Sanger sequencing to test the M-ES Tn mutants for point mutations in PDIM biosynthesis genes that we identified in other Tn mutants from our library. This analysis identified 6 M-ES Tn mutants with mutations in genes required for PDIM production (**Tab B in S1 Table**, unusable Tn mutants). We conducted whole genome sequencing on the remaining M-ES Tn mutants, which identified additional mutations predicted to prevent PDIM production in 17 Tn mutants and one mutant with multiple Tn insertions (**Tab B in S1 Table**, unusable Tn mutants). These mutants were excluded from further analysis. We recovered Tn mutants in 19 M-ES genes which contained a single Tn insertion and no mutations in genes required for PDIM production, based on whole genome sequencing (**Tab A in S1 Table**, Tn mutants in M-ES library). These M-ES Tn mutants, representing various metabolic pathways and essential processes (**Table 1**), were selected for analysis of growth *in vitro* and fitness defects in mice.

**Table 1:** Tn mutants in M-ES genes in the Tn library screened in mice.

Gene	Annotation	Putative Function
*purF*	amidophosphoriboysltransferase	*de novo* purine & thiamine biosynthesis
*purM*	phosphoribosyl-aminoimidazole synthase	*de novo* purine & thiamine synthesis
*purQ*	phosphoribosyl-formylglycinamidine synthetase I	*de novo* purine & thiamine synthesis
*pheA*	prephenate dehydratase	Phe biosynthesis
*trpG*	anthranilate synthase component II	Trp biosynthesis
*trpB*	tryptophan synthase beta chain	Trp biosynthesis
*proC*	pyrroline-5-carboxylate reductase	Pro biosynthesis
*gltB*	ferredoxin-dependent glutamate synthase	Glu biosynthesis
*ilvA*	threonine dehydratase	Ile biosynthesis
*panC*	pantoate-beta-alanine ligase	pantothenate biosynthesis
*ribA2*	GTP cyclohydrolase II	riboflavin biosynthesis
*ribG*	bifunctional riboflavin synthesis protein	riboflavin biosynthesis
*pabB*	*para*-aminobenzoic acid (PABA) synthase component I	PABA biosynthesis
*fhaA*	conserved protein with forkhead-associated domain	regulation of cell wall synthesis
*pstP*	serine-threonine phosphatase	regulation of cell wall synthesis
*ftsQ*	cell division protein	cell division
*ERDMAN_3321*	possible acyl transferase	unknown function
*ERDMAN_2739*	Obg GTPase	ribosome assembly
*ERDMAN_4254*	LigA DNA ligase	DNA damage repair

### Tn mutants in central metabolic pathways have growth defects in Middlebrook 7H9 medium

**Fig 1:**
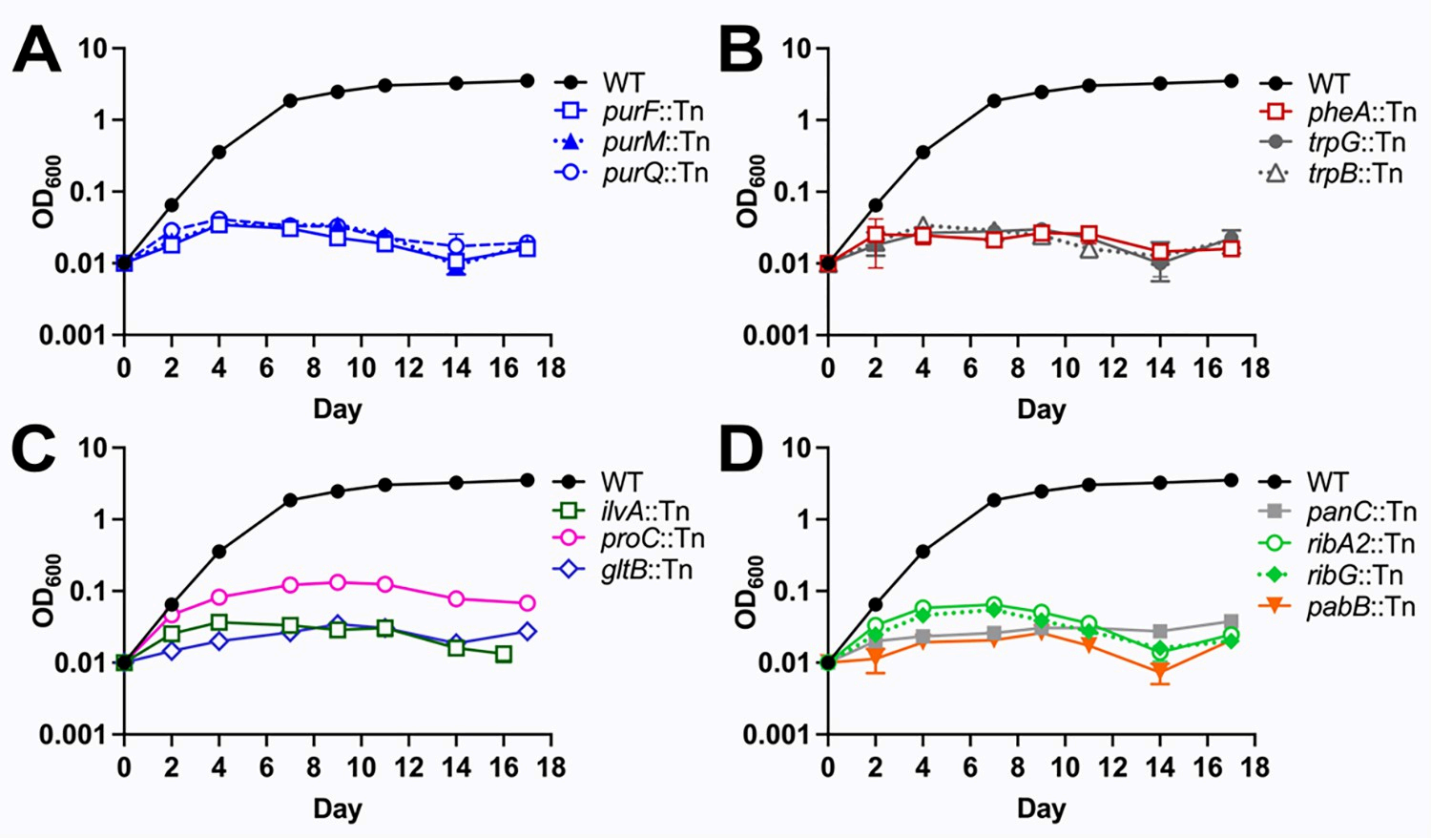
*M*. *tuberculosis* Tn mutants in genes encoding central metabolic enzymes exhibit growth defects in Middlebrook 7H9 medium. Wild-type *M*. *tuberculosis* Erdman and M-ES Tn mutant strains were grown in MtbYM rich medium, washed twice in PBS-T, and diluted to OD_600_ = 0.01 in Middlebrook 7H9 medium. Growth was monitored by measuring the OD_600_. Tn mutants are grouped according to predicted function: **(A)** purine and thiamine metabolism (*purF*, *purM*, *purQ*), **(B-C)** amino acid biosynthesis (*pheA*, *trpB*, *trpG*, *proC*, *gltB*, *ilvA*), **(D)** pantothenate, riboflavin, and *p-*amino benzoic acid (PABA) biosynthesis (*panC*, *ribA2*, *ribG*, *pabB)*. Data represent the mean ± standard error of three biological replicates.

To determine whether the M-ES genes disrupted by Tn insertion are required for *M*. *tuberculosis* growth in standard culture conditions, we conducted growth curves in Middlebrook 7H9 medium. Tn mutants in genes encoding various central metabolic pathway enzymes exhibited severe growth defects. These included Tn mutants in genes required for purine and thiamine biosynthesis (*purF*, *purM*, *purQ*; **Fig 1A**), biosynthesis of the amino acids Phe, Trp, Pro, Glu and Ile (*pheA*, *trpB*, *trpG*, *proC*, *gltB*, *ilvA*; **Fig 1B and 1C**), and riboflavin or *para*-amino benzoic acid (PABA) biosynthesis (*ribA2*, *ribG*, *pabB*; **Fig 1D**). The *panC*::Tn mutant was included in our study as a positive control, and as expected it also failed to grow in Middlebrook 7H9 (**Fig 1D**). *M*. *tuberculosis* requires PanC for synthesis of pantothenate (vitamin B5), an essential precursor in coenzyme A biosynthesis, in the absence of exogenous pantothenate [29]. These data demonstrate that each of these M-ES genes is essential for growth of *M*. *tuberculosis* Erdman in standard Middlebook medium, consistent with their annotation as essential for growth on Middlebrook 7H10 based on saturating Tn mutagenesis [11].

Tn mutants in six other putative M-ES genes had no growth defects in Middlebrook 7H9 medium (**S1 Fig**). Four Tn mutants harbored insertions in non-essential domains of M-ES genes encoding a putative acetyl transferase (*ERDMAN_3321*::Tn), regulators of cell wall synthesis (*pstP*::Tn, *fhaA*::Tn) and a regulator of cell division (*ftsQ*::Tn) [11,30–32]. Two other Tn mutants were in misannotated *M*. *tuberculosis* Erdman genes. *ERDMAN_2739* is annotated as *obg*, an essential GTPase that controls ribosome assembly [11,33–35], but encodes PE_PGRS43, which is not essential. *ERDMAN_4254* is annotated as a DNA ligase, with possible similarity to the essential DNA ligase *ligA* (*rv3014*) [11,35], but encodes the non-essential ESX-1 substrate EspK [36]. Thus, a subset of the Tn mutants we isolated do not disrupt functions essential for *M*. *tuberculosis* growth in Middlebrook medium.

### *M*. *tuberculosis* requires many central metabolic pathways for fitness in mice

**Fig 2:**
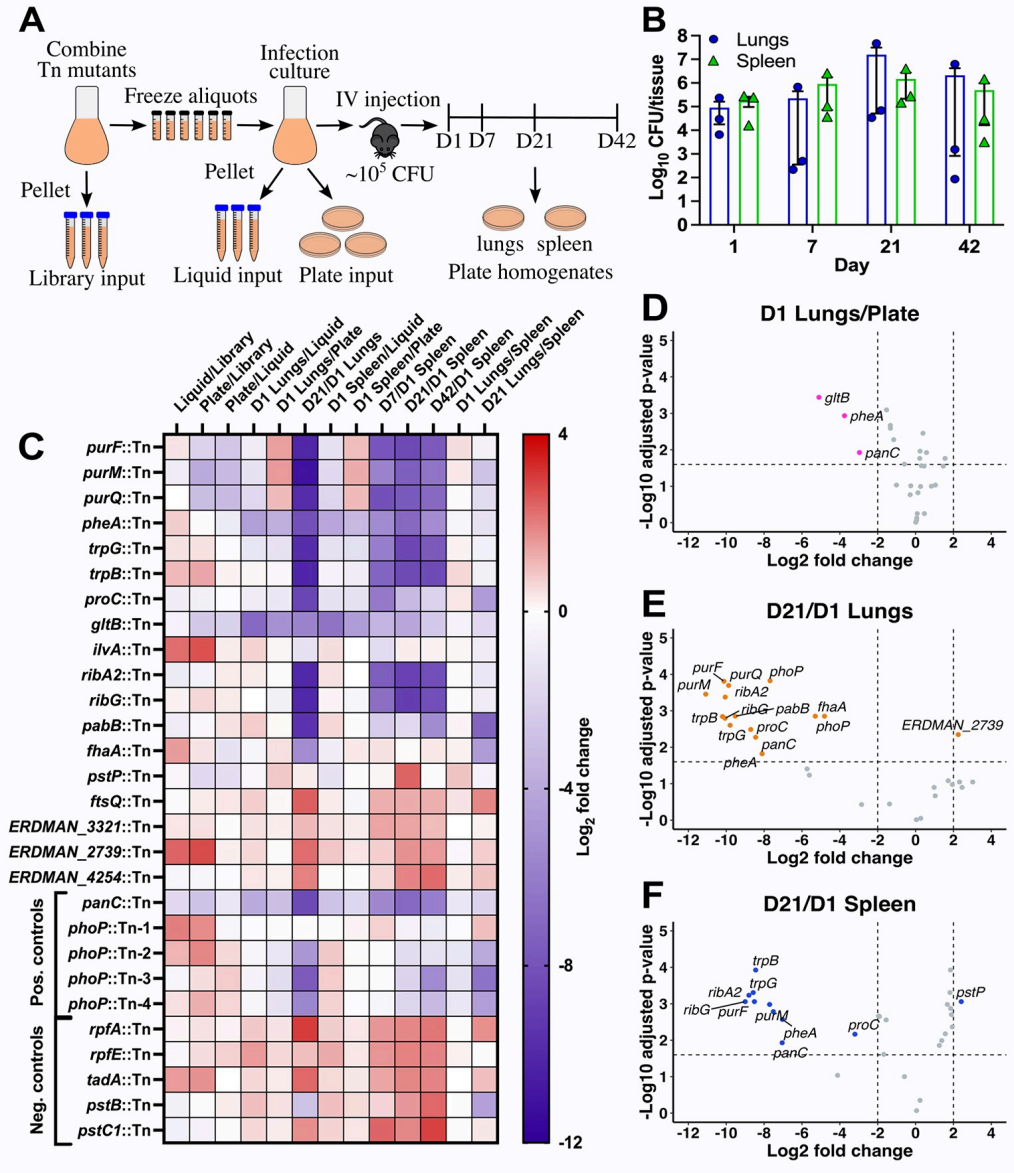
Tn-seq screen of the *M*. *tuberculosis* M-ES Tn library identifies multiple central metabolic pathways required for fitness in mice. **(A)** M-ES Tn-seq screen methods. Individual Tn mutants grown in MtbYM rich medium were mixed in equal abundance to create the M-ES Tn library. Triplicate M-ES Tn library samples were collected for Tn-seq (Library control) and the remainder was aliquoted and frozen for experiments. The M-ES Tn library was grown in MtbYM rich broth, then washed and diluted in PBS-T to OD_600_ = 0.05. Triplicate samples of the diluted M-ES Tn library were collected (Liquid control) and the diluted M-ES Tn library was plated on MtbYM rich agar in triplicate (Plate control) for Tn-seq. Mice were injected via the lateral tail vein with ~10^5^ CFU of the M-ES Tn library to seed at least 10^4^ CFU in the lungs and spleen. Mice (*n* = 3) were euthanized at days 1, 7, 21, and 42. Lung and spleen homogenates were plated on MtbYM rich agar to recover surviving Tn mutants for Tn-seq. **(B)** *M*. *tuberculosis* CFU recovered from lungs (blue) and spleens (green). **(C)** Heat map of Log_2_ fold change in Tn mutant abundance between the indicated conditions determined by TnseqDiff analysis of Tn-seq data. Positive values (red) indicate a relative fitness advantage; negative values (blue) indicate a relative fitness defect. **(D-F)** Volcano plots of TnseqDiff analysis of Tn-seq data for the M-ES Tn mutant library for **(D)** lungs at day 1 compared to the Plate control; **(E)** lungs at day 21 compared to lungs at day 1; or **(F)** spleens at day 21 compared to spleens at day 1. Dashed lines indicate cutoffs for statistical significance of ± 2 Log_2_ fold change and adjusted *P* value <0.025. Tn mutants meeting these significance cutoffs are colored and labeled.

To identify those M-ES Tn mutants with fitness defects in mice, we created and screened a library of Tn mutants in M-ES genes (**Tab A in S1 Table**). We included in the M-ES Tn library five positive control Tn mutants in genes known to be required for *M*. *tuberculosis* fitness in mice: *panC*::Tn [29] and four independent Tn insertions in *phoP*, which encodes a response regulator essential for virulence [37,38]. We also included five negative control Tn mutants in genes known to be dispensable for *M*. *tuberculosis* growth and survival in mice: *rpfA* and *rpfE* that encode redundant resuscitation promoting factors [39], *pstB* and *pstC1* that encode subunits of an alternate Pst phosphate transporter [40], and *tadA* that encodes a Flp pilin assembly ATPase [41]. Each of these negative control Tn mutants were predicted to be PDIM-proficient based on whole genome sequencing (**Tab A in S1 Table**). The Tn mutants were grown individually in MtbYM rich liquid medium and equivalent amounts of each mutant were combined to generate the M-ES Tn library (**Fig 2A**). Bacteria in the M-ES Tn library were collected in triplicate for genomic DNA extraction as a control to confirm equal representation of each Tn mutant and aliquots of the library were frozen for use in experiments (**Fig 2A**).

To screen the M-ES Tn library for fitness defects in mice, the library was grown in MtbYM rich liquid, pelleted in triplicate for genomic DNA extraction as an input control (Liquid control), plated on MtbYM rich agar as a control for recovery on plates (Plate control), and used to infect C57BL/6J mice by intravenous injection (**Fig 2A**). While this is not the natural infection route, intravenous injection was necessary to overcome the bottleneck to infection by the aerosol route and ensure adequate representation of all Tn mutants in the lungs. The intravenous route also enabled analysis of Tn mutant fitness in both lung and spleen tissues, which we expected would not be possible by the aerosol route as we predicted that many M-ES Tn mutants would be strongly attenuated in lungs and unable to disseminate to the spleen. At days 1, 7, 21 and 42 post-infection, groups of mice (*n* = 3) were euthanized and surviving *M*. *tuberculosis* CFU were recovered from lung and spleen homogenates by plating on MtbYM rich agar (**Fig 2A**). We noted high variability in *M*. *tuberculosis* CFU recovered from the lungs at days 7 and 42 (**Fig 2B**). For analysis of Tn mutant fitness by Tn-seq, we selected plates containing at least 10^4^ *M*. *tuberculosis* CFU for genomic DNA extraction. Since only one mouse each at the day 7 and day 42 time points had sufficient CFU in the lungs for Tn-seq analysis (**Fig 2B**), the lung samples were analyzed only at days 1 and 21. Sufficient bacteria were recovered from the spleens at all time points for Tn-seq analysis (**Fig 2B**).

To determine Tn mutant fitness, the triplicate genomic DNA samples from the library input, liquid input, plate input, and tissue homogenates plated on MtbYM rich agar (**Fig 2A**) were subjected to Tn-seq analysis and read counts for each Tn insertion site were tabulated (**S2 Table**). To determine relative Tn mutant fitness, we used TnseqDiff, which identifies conditionally essential genes between conditions based on the relative frequencies of Tn-seq sequencing reads at each Tn insertion site [42]. We previously used this approach to identify Tn mutants with altered fitness in low-complexity *M*. *tuberculosis* Tn mutant libraries treated with antibiotics *in vitro* [28]. Complete TnseqDiff analyses are provided in **S3 Table**.

No statistically significant fitness defects were observed in comparisons between the original M-ES Tn library and the MtbYM rich liquid culture that was used to infect the mice (**Figs 2C and S2A**), suggesting that all Tn mutants grow equally well in MtbYM rich medium. In TnseqDiff comparisons between the original M-ES Tn library and the input plated on MtbYM rich agar, several M-ES Tn mutants showed slight, but statistically significant fitness defects (*purF*::Tn, *purM*::Tn, *purQ*::Tn, *gltB*::Tn, *panC*::Tn; **Figs 2C and S2B**). These Tn mutants may have growth defects on MtbYM rich agar compared to other mutants in the M-ES Tn library, which causes their under-representation after recovery on plates. These data suggest that most M-ES Tn mutants are efficiently recovered on MtbYM rich agar and that more pronounced fitness defects observed following mouse infection are due to inability to survive within the mouse rather than a strong competitive disadvantage on MtbYM rich agar.

To determine relative Tn mutant fitness at day 1 post-infection, we compared Tn mutant abundance in plated mouse tissues to the input plated on MtbYM agar (D1 lungs/plate or D1 spleen/plate) using TnseqDiff. Three Tn mutants (*gltB*::Tn, *pheA*::Tn, *panC*::Tn) were significantly under-represented in the lungs at day 1 post-infection (**Fig 2C and 2D**). Attenuation of *panC*::Tn was expected, as *panC* was essential for *M*. *tuberculosis* growth in BALB/c mice [29]. We observed similarly reduced fitness of the *gltB*::Tn and *pheA*::Tn mutants in the spleen at day 1 post-infection (**Figs 2C and S2C**). These data suggest that *M*. *tuberculosis* requires Phe biosynthesis (PheA) and Glu biosynthesis (GltB) for survival in the host at the earliest stage of infection, possibly to counteract limited availability of these amino acids within infected phagocytes.

To determine Tn mutant fitness in the lungs and spleens at later times post-infection, the day 7, day 21 or day 42 outputs were compared to the day 1 input from the corresponding tissue using TnseqDiff. We identified 14 Tn mutants that exhibited significantly reduced fitness in the lungs at day 21 post-infection (**Fig 2C and 2E**). These included Tn mutants in genes encoding enzymes required for purine and thiamine biosynthesis (*purF*::Tn, *purM*::Tn, *purQ*::Tn), amino acid biosynthesis (*pheA*::Tn, *trpB*::Tn, *trpG*::Tn, *proC*::Tn), riboflavin synthesis (*ribA2*::Tn, *ribG*::Tn) and PABA synthesis (*pabB*::Tn), as well as the *panC*::Tn and *phoP*::Tn positive controls. Attenuation of the *trpB*::Tn and *trpG*::Tn mutants was expected as another gene in the Trp biosynthesis pathway (*trpE*) was previously implicated in *M*. *tuberculosis* growth in C57BL/6 mice [13].

We observed a similar pattern of attenuation in the spleens at day 21. Most Tn mutants that were attenuated in lungs also exhibited significant fitness defects in the spleen (**Fig 2C and 2F**). One exception is *pabB*::Tn, which was significantly attenuated in lungs but not spleens (**Fig 2C, 2E and 2F**). The Tn mutants that exhibited significant fitness defects in the spleen at day 21 were also significantly attenuated in the spleen at days 7 and 42 post-infection (**Figs 2C, S2D, and S2E**). The *pheA*::Tn mutant was significantly attenuated in lungs and spleens at all time points, despite significantly reduced fitness at day 1 (**Fig 2C**). In contrast, the *gltB*::Tn mutant was attenuated in lungs and spleens, but this did not achieve statistical significance, possibly because the *gltB*::Tn mutant was at much lower abundance in the day 1 normalization controls (**Figs 2C and S2C**). Overall, these data suggest that *M*. *tuberculosis* requires multiple central metabolic pathways, particularly purine nucleotide and thiamine biosynthesis, riboflavin biosynthesis, and biosynthesis of specific amino acids (Phe, Trp, Pro, Glu) to replicate within host tissues.

While most M-ES Tn mutants with *in vitro* growth defects were attenuated in mice, the *ilvA*::Tn mutant was a notable exception. The *ilvA*::Tn mutant did not replicate in Middlebrook 7H9 *in vitro* (**Fig 1C**), but exhibited no statistically significant fitness defects in mouse lungs or spleens at any time point (**Fig 2C**). These data suggest that *M*. *tuberculosis* does not require IlvA for Ile synthesis during mouse infection.

### Purine and thiamine auxotrophy is cidal for *M*. *tuberculosis in vitro* and in mice

**Fig 3:**
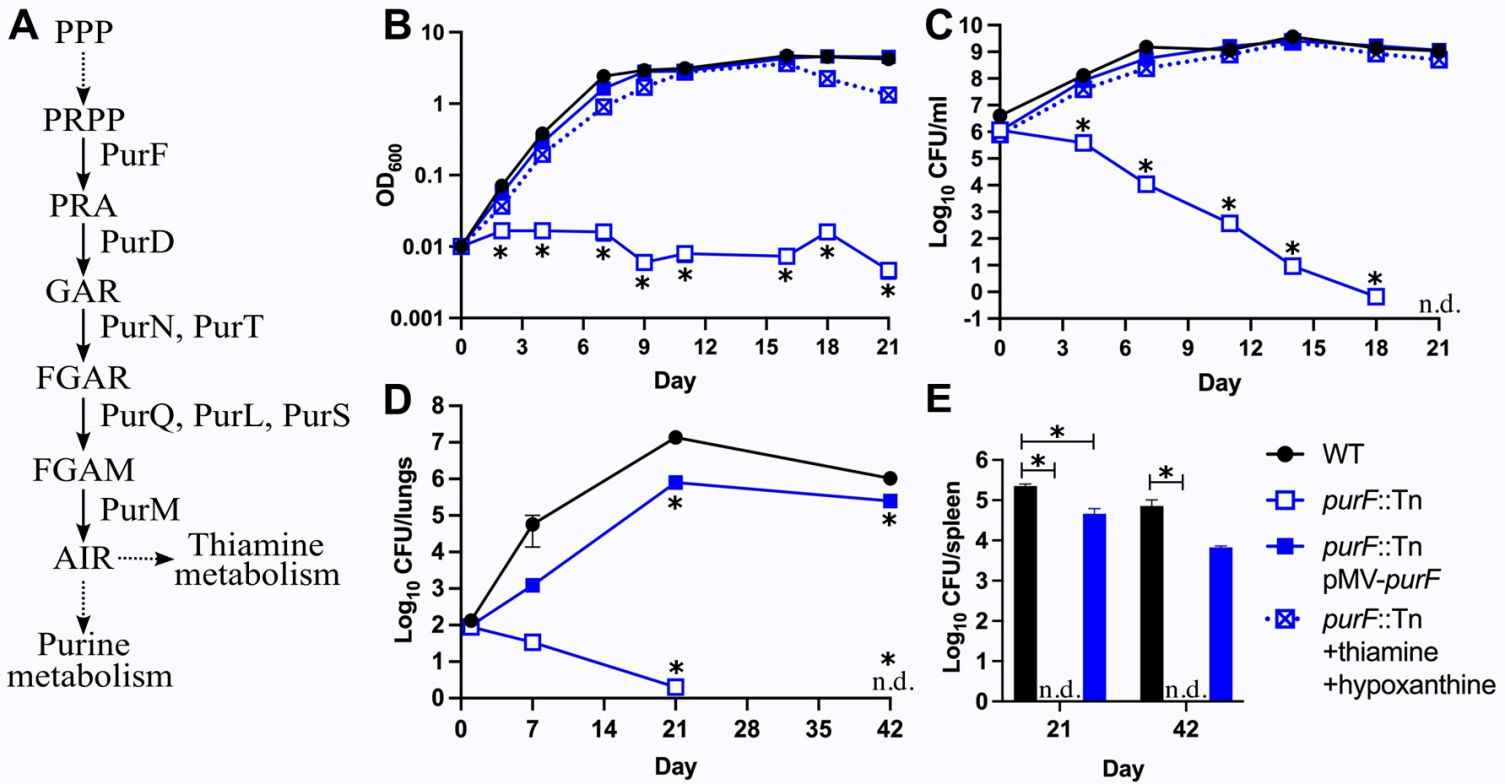
Loss of *purF* causes death of *M*. *tuberculosis* in the absence of nutrient supplementation *in vitro* and in mice. **(A)** Pathway for *de novo* synthesis of purine nucleotides and thiamine in *M*. *tuberculosis*. Solid lines indicate a single step; dashed lines indicate multiple steps. Proteins that catalyze the first five steps are indicated. Intermediate abbreviations: PPP, pentose phosphate pathway; PRPP, 5-phosphoribosyl pyrophosphate; PRA, 5-phospho-D-ribosylamine; GAR, 5’-phosphoribosylglycinamide; FGAR, 5’-phosphoribosyl-N-formylglycinamide; FGAM, 5’-phosphoribosyl N-formylglycinamidine; AIR, 5-aminoimidazole ribotide. **(B-C)** Strains grown in 7H9 supplemented with 60 μM thiamine and 150 μM hypoxanthine were washed in PBS-T and diluted to OD_600_ = 0.01 in 7H9 or 7H9 supplemented with 60 μM thiamine and 150 μM hypoxanthine. Bacterial growth and survival were measured by **(B)** optical density at 600 nm or **(C)** serial dilutions and plating to recover viable CFU. **(D-E)** C57BL/6J mice were infected by aerosol with the indicated strains. Mice (*n* = 6) were euthanized at days 1, 7, 21, and 42 post-infection. Lung **(D)** and spleen **(E)** homogenates were serially diluted and plated to recover viable CFU. In **(C-E)**, WT and *purF*::Tn pMV-*purF* were plated on 7H10 agar; *purF*::Tn was plated on 7H10 agar supplemented with 60 μM thiamine and 150 μM hypoxanthine. Data represent the mean ± standard error of three biological replicates **(B-C)** or six animals **(D-E)**. Asterisks indicate *P*-value < 0.05; n.d. indicates not detected (detection limit = 1 CFU).

To confirm the results of our Tn-seq screen, we selected three Tn mutants for individual retesting. We selected *purF*::Tn as a representative mutant with defects in biosynthesis of purine and thiamine. PurF catalyzes the first committed step of *de novo* purine nucleotide biosynthesis, which also produces a metabolic precursor required for the *de novo* synthesis of thiamine (vitamin B1) (**Fig 3A**) [43]. In *M*. *tuberculosis*, purine nucleotides can be produced either *de novo* or via a salvage pathway from hypoxanthine, which is converted to the purine nucleotide precursor inosine monophosphate (IMP) by hypoxanthine-guanine phosphoribosyltransferase (Hpt) [44].

We confirmed that the *purF*::Tn mutant fails to grow in standard Middlebrook 7H9 medium and that the growth defect can be alleviated with exogenous hypoxanthine and thiamine (**Fig 3B**), the two components of MtbYM rich predicted to bypass *de novo* purine biosynthesis [27]. Exogenous hypoxanthine alone partially rescued growth of the *purF*::Tn mutant, but full growth restoration only occurred with both nutrients added (**S3 Fig**). The growth defect of the *purF*::Tn mutant in 7H9 was also fully complemented by *purF* expressed from a plasmid (pMV-*purF*, **Fig 3B**). The *purF*::Tn mutant lost viability in unsupplemented 7H9, with viable CFU below the limit of detection (1 CFU/ml) after 21 days of incubation (**Fig 3C**). Viability of the *purF*::Tn mutant was restored by either exogenous hypoxanthine and thiamine or by complementation with pMV-*purF* (**Fig 3C**). These data demonstrate that loss of PurF function causes rapid sterilization of *M*. *tuberculosis* in the absence of nutrient supplementation.

To determine if *M*. *tuberculosis* requires PurF during infection, we infected C57BL/6J mice by aerosol with WT Erdman, *purF*::Tn or the *purF*::Tn pMV-*purF* complemented strain. All strains were confirmed to produce the PDIM lipid that is essential for virulence using thin layer chromatography of ^14^C-propionate labelled lipid extracts (**S4 Fig**). The *purF*::Tn mutant established infection in the lungs at day 1 post-infection, but it failed to replicate, was cleared from the lungs by day 42 post-infection, and failed to disseminate to the spleen (**Fig 3D and 3E**). Attenuation of the *purF*::Tn mutant was partially complemented by pMV-*purF*. The *purF*::Tn pMV-*purF* strain replicated in the lungs and disseminated to spleen, but significantly fewer CFU were recovered compared to the WT control (**Fig 3D and 3E**). Expression of *purF* from the pMV-*purF* plasmid may be insufficient to support *M*. *tuberculosis* growth in host tissues. Alternatively, the *purF*::Tn insertion may be polar on the 3’ *purM* gene, which is also a M-ES gene [11]. Reduced expression of *purF* or *purM* could prevent normal *M*. *tuberculosis* replication in mice, in which demand for purine nucleotides and/or thiamine may be higher as compared to *in vitro* culture. Overall, our data demonstrate that the Tn insertion in *purF*, not a secondary mutation, causes attenuation in mice. These data indicate that *M*. *tuberculosis* cannot obtain sufficient purine nucleotide precursors and/or thiamine to support growth in host tissues and implicate the purine and thiamine biosynthetic pathways as TB drug targets.

### *M*. *tuberculosis* requires Phe biosynthesis for survival *in vitro* and in mice

**Fig 4:**
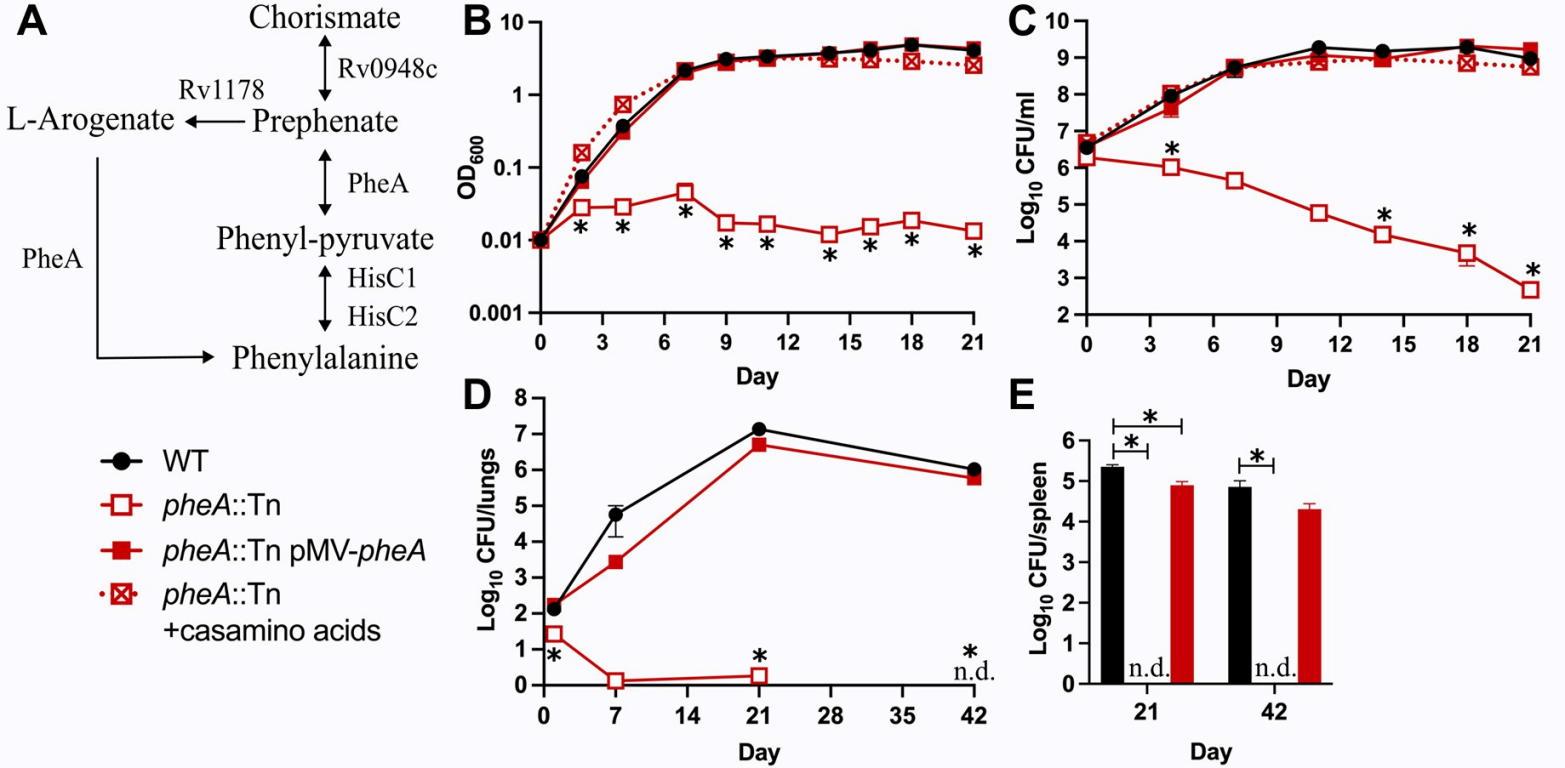
*M*. *tuberculosis* requires Phe synthesis for survival *in vitro* in the absence of amino acid supplementation and in mice. **(A)** Phenylalanine biosynthesis in *M*. *tuberculosis*. Single arrows represent one-way reactions. Double arrows represent reversible reactions. Proteins predicted to catalyze each step are indicated. **(B-C)** Strains grown in 7H9 supplemented with 0.5% casamino acids were washed in PBS-T and diluted to OD_600_ = 0.01 in 7H9 or 7H9 supplemented with 0.5% casamino acids. Bacterial growth and survival were measured by **(B)** optical density at 600 nm and **(C)** serial dilutions and plating to recover viable CFU. **(D-E)** C57BL/6J mice were infected by aerosol with the indicated strains. Mice (*n* = 6) were euthanized at days 1, 7, 21, and 42 post-infection. Lung **(D)** and spleen **(E)** homogenates were serially diluted and plated to recover viable CFU. In **(C-E)**, WT and *pheA*::Tn pMV-*pheA* were plated on 7H10 agar; *pheA*::Tn was plated on 7H10 agar with 0.5% casamino acids. Data represent the mean ± standard error of three biological replicates **(B-C)** or six animals **(D-E)**. Asterisks indicate *P*-value < 0.05; n.d. indicates not detected (detection limit = 1 CFU).

We selected the *pheA*::Tn mutant for retesting as the role of Phe biosynthesis in *M*. *tuberculosis* fitness either *in vitro* or in the host has not previously been tested and the Phe biosynthesis pathway is absent in humans, making it an attractive drug target [45]. PheA is a prephenate dehydratase required for both pathways of Phe synthesis (**Fig 4A**). PheA catalyzes the first step in Phe synthesis from prephenate [46], which is produced from chorismate by chorismate mutase [45]. Two *M*. *tuberculosis* proteins have chorismate mutase activity (Rv1885c and Rv0948c), but Rv1885c is secreted while Rv0948c is cytosolic [47–49]. Only *rv0948c* is a M-ES gene [11], suggesting that it functions as the chorismate mutase in Phe synthesis. In plants and some bacteria, Phe synthesis can also proceed via an L-arogenate intermediate [45]. L-arogenate has been detected in *M*. *tuberculosis* [50], and the aminotransferase Rv1178 may synthesize L-arogenate from chorismate, as this enzyme activity has been demonstrated *in vitro* [51]. PheA is predicted to act as the dehydratase to convert L-arogenate to Phe.

The *pheA*::Tn mutant does not grow in 7H9 medium unless the medium is supplemented with casamino acids or with Phe (**Figs 4B and S5A**), confirming that it is a Phe auxotroph. The growth defect of the *pheA*::Tn mutant in 7H9 was also fully complemented with plasmid-encoded *pheA* (pMV-*pheA*, **Fig 4B**). The *pheA*::Tn mutant lost viability in 7H9 in the absence of casamino acid supplementation, indicating that an inability to acquire or produce Phe is cidal to *M*. *tuberculosis* (**Fig 4C**). This survival defect was fully complemented by pMV-*pheA* (**Fig 4C**). These data demonstrate that blocking Phe synthesis causes death of *M*. *tuberculosis* in the absence of exogenous nutrient supplementation.

To confirm attenuation of the *pheA*::Tn mutant, C57BL/6J mice were infected by aerosol with WT Erdman, *pheA*::Tn, or the *pheA*::Tn pMV-*pheA* complemented strain. The *pheA*::Tn and *pheA*::Tn pMV-*pheA* strains both produce the PDIM lipid (**S4 Fig**). Despite using a higher inoculating dose, we observed significantly fewer *pheA*::Tn bacteria in the lungs at 24 hr post-infection compared to either the WT or complemented controls (**Fig 4D**). This is consistent with the significant reduction in fitness we observed for the *pheA*::Tn mutant at day 1 post-infection compared to the plated input control in our Tn-seq screen (**Fig 2C and 2D**). These data suggest that PheA is required for *M*. *tuberculosis* to establish infection in the lungs. The *pheA*::Tn mutant was also incapable of replicating in mouse lungs, was cleared to below the limit of detection from lung tissues by 42 days post-infection, and failed to disseminate to the spleen (**Fig 4D and 4E**). The *pheA*::Tn pMV-*pheA* complemented strain replicated in the lungs (**Fig 4D**) and disseminated to spleen (**Fig 4E**) similarly to the WT control. These data show that attenuation of the *pheA*::Tn mutant is due to the Tn insertion in *pheA* and not a secondary mutation. These data also demonstrate that *M*. *tuberculosis* requires Phe biosynthesis to survive within the host, suggest that Phe is not available in sufficient quantities in host tissues to support *M*. *tuberculosis* growth, and indicate that PheA is a potential target for development of new TB drugs.

### *M*. *tuberculosis* does not require IlvA during infection

**Fig 5:**
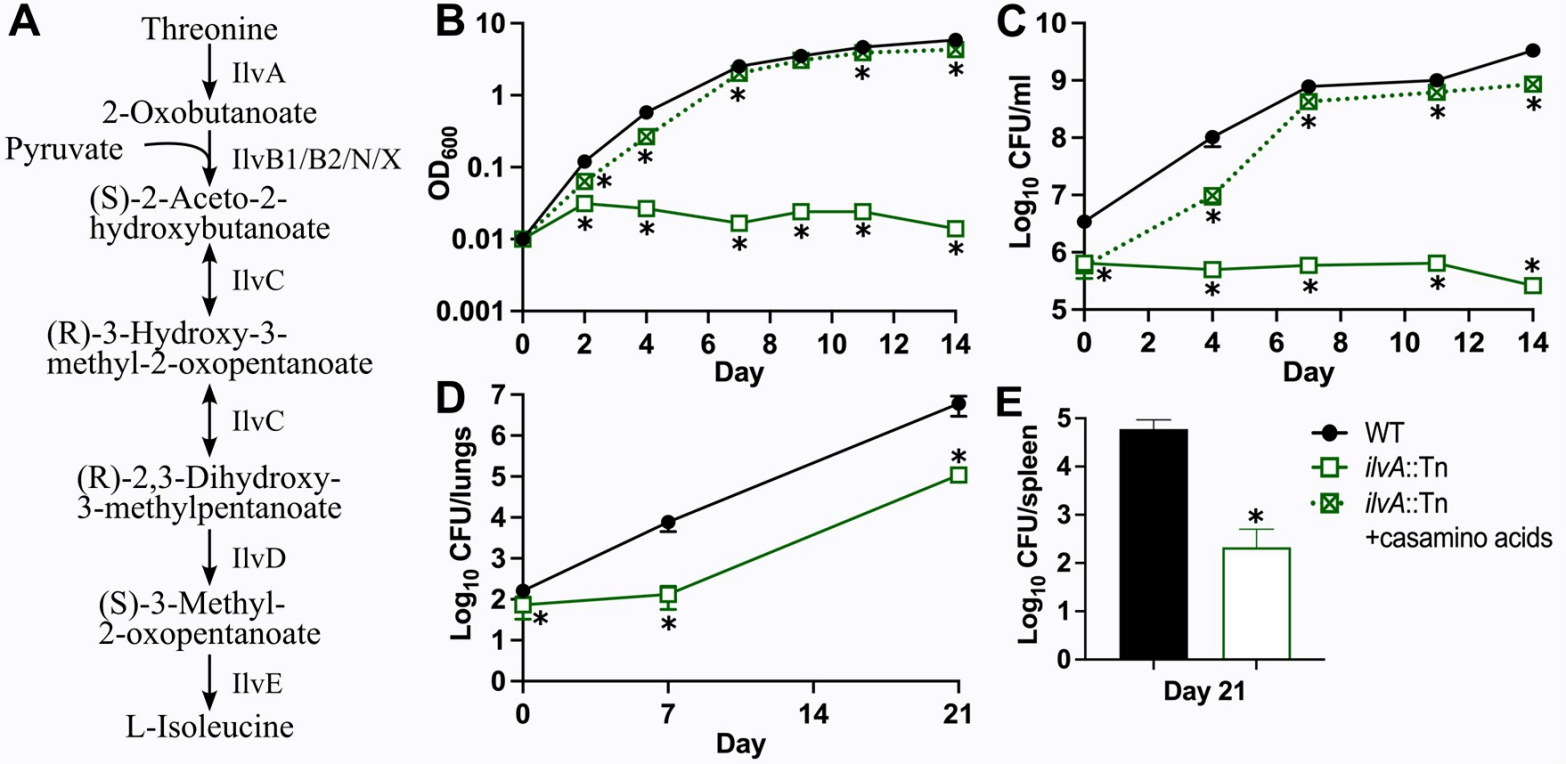
*M*. *tuberculosis* does not require IlvA in mice. **(A)** Isoleucine biosynthesis in *M*. *tuberculosis*. Single arrows represent one-way reactions. Double arrows represent reversible reactions. Proteins predicted to catalyze each step are indicated. **(B-C)** Strains grown in 7H9 supplemented with 0.5% casamino acids were washed in PBS-T and diluted to OD_600_ = 0.01 in 7H9, or 7H9 with 0.5% casamino acids. Bacterial growth and survival were measured by **(B)** optical density at 600 nm and **(C)** serial dilutions and plating to recover viable CFU. **(D-E)** C57BL/6J mice were infected by aerosol with WT Erdman or *ilvA*::Tn. Mice (*n* = 6) were euthanized at days 1, 7 and 21 post-infection. Lung **(D)** and spleen **(E)** homogenates were serially diluted and plated to recover viable CFU. In **(C-E)** WT was plated on 7H10 agar; *ilvA*::Tn was plated on 7H10 agar with 0.5% casamino acids. Data represent the mean ± standard error of three biological replicates **(B-C)** or six animals **(D-E)**. Asterisks indicate *P*-value <0.05.

Finally, we selected the *ilvA*::Tn mutant for individual retesting to confirm the results of our screen, which suggested that *M*. *tuberculosis* does not require IlvA for growth in mice. IlvA catalyzes the first committed step in Ile synthesis, converting threonine to 2-oxobutanoate (2-ketobutyrate) (**Fig 5A**), and is uniquely required for Ile synthesis [52,53]. Some bacteria produce 2-oxobutanoate by an alternative route using pyruvate and acetyl-CoA via a citramalate intermediate [54]. However, *M*. *tuberculosis* cannot use this alternative route to synthesize Ile, at least *in vitro*, since the *ilvA*::Tn mutant is an Ile auxotroph. The *ilvA*::Tn mutant does not grow in Middlebrook 7H9 medium unless it is supplemented with casamino acids or Ile (**Figs 5B and S5B**). However, the *ilvA*::Tn mutant remained viable without amino acid supplementation *in vitro*, suggesting that Ile starvation is not cidal for *M*. *tuberculosis* (**Fig 5C**). We confirmed that the *ilvA*::Tn mutant produces PDIM (**S4 Fig**). In C57BL/6J mice infected by aerosol, the *ilvA*::Tn mutant replicated in the lungs, albeit with slower kinetics than WT Erdman (**Fig 5D**). By 3 weeks post-infection, the *ilvA*::Tn mutant had also disseminated to the spleen (**Fig 5E**). These data indicate that while *M*. *tuberculosis* requires IlvA to synthesize Ile *in vitro*, it can either acquire sufficient Ile from the host or bypass IlvA function via an alternative Ile synthesis route during infection.

## Discussion

*M*. *tuberculosis* requires many metabolic pathways for growth in standard *in vitro* conditions, but whether these pathways are also required during infection has not been comprehensively evaluated. We used a Tn mutant collection generated using a nutrient-enriched medium to demonstrate that several metabolic pathways essential for growth in standard Middlebook medium are also essential during mouse infection. Our data show that *M*. *tuberculosis* requires *de novo* purine and thiamine biosynthesis as well as Phe biosynthesis to replicate in the lungs and disseminate to the spleen in aerosol-infected mice, suggesting that *M*. *tuberculosis* cannot acquire these nutrients in sufficient quantities from host tissues. In addition, we show that an inability to synthesize Phe or purine and thiamine causes death of *M*. *tuberculosis* without exogenous nutrients, suggesting that inhibitors of these biosynthetic pathways would be bactericidal. Our data implicate the Phe and purine/thiamine biosynthesis pathways as promising targets for development of novel anti-tubercular drugs.

*M*. *tuberculosis* Phe biosynthesis has been explored as a drug target because mammals cannot synthesize Phe, which is expected to limit toxicity of drugs targeting this pathway [45]. *M*. *tuberculosis* imports all amino acids, including Phe [55], which we confirm by rescuing growth of the *pheA*::Tn mutant with exogenous Phe. Isotopic labeling revealed that *M*. *tuberculosis* acquires Phe from infected macrophages, but computational modeling of nitrogen flux indicated that Phe import cannot support the biomass requirement [56]. Our data showing that *M*. *tuberculosis* requires PheA during mouse infection are consistent with this model and with the observation that *pheA* transcriptional inhibition using CRISPR interference (CRISPRi) caused attenuation in macrophages [57]. A genome-wide CRISPRi screen also identified *pheA* as a gene highly vulnerable to inhibition *in vitro*, comparable to genes encoding the targets of existing anti-tubercular drugs [9], suggesting Phe synthesis inhibitors could be effective antibiotics. Inhibitors of *M*. *tuberculosis* chorismate mutase, which is also required for Phe synthesis [45], have been identified. However, most of these compounds had high minimal inhibitory concentrations (MICs), suggesting they have low whole cell permeability [58–60]. Since *M*. *tuberculosis* imports Phe, it may be possible to design PheA or chorismate mutase inhibitors with improved permeability properties.

Purine nucleotide biosynthesis has also been explored for antibiotic development since many bacterial pathogens require this pathway for virulence and nucleotide synthesis inhibitors, which include approved cancer chemotherapeutics, have antibiotic activity [61]. Since *de novo* purine biosynthesis includes 10 enzymatic steps, it represents a pathway rich in targets for anti-tubercular drug development. Mammals also synthesize purine nucleotides *de novo*, but the mycobacterial PurN, PurH, PurC and PurF enzymes are structurally distinct from the human orthologs, suggesting that specific inhibitors can be designed [43,62–65]. Our data demonstrate that *M*. *tuberculosis* requires PurF, which catalyzes the first committed step of *de novo* purine nucleotide and thiamine co-factor biosynthesis, for growth in the host. Our data suggest that *M*. *tuberculosis* also requires other enzymes in the purine and thiamine biosynthetic pathways during infection, as *purM*::Tn and *purQ*::Tn mutants were also highly attenuated in our screen. Our data are consistent with a report that a *M*. *tuberculosis purC* mutant was cleared from intravenously-infected mice [66] and with the observation that depletion of GuaB2, which is required for *de novo* guanine nucleotide synthesis, prevents *M*. *tuberculosis* replication in aerosol-infected mice [67]. Collectively, our data indicate that *M*. *tuberculosis* cannot acquire sufficient purine nucleotides and/or thiamine from host tissues, and support development of inhibitors targeting these pathways.

Inhibitors targeting *M*. *tuberculosis* purine nucleotide and thiamine synthesis have been described. Compounds that inhibit *Mycobacterium abscessus* PurC also prevented *M*. *tuberculosis* growth *in vitro* [65]. A GuaB2 inhibitor was cidal against replicating *M*. *tuberculosis in vitro* but lacked activity in chronically infected mice due to either low drug concentration in host tissues, slower growth of *M*. *tuberculosis* in mice, or high levels of guanine in host tissues that antagonize the drug [68]. The *de novo* thiamine biosynthesis pathway is also a promising target for antimicrobial development because it is absent in humans, who must obtain thiamine in the diet [69]. An inhibitor of *M*. *tuberculosis* ThiE, which catalyzes the final step in production of the active co-enzyme thiamine pyrophosphate, potently inhibited *in vitro* growth [70]. Compounds that inhibit one of the first five steps in purine biosynthesis, which would prevent both purine and thiamine production, may prove to be most effective because they would block multiple metabolic functions.

Our data suggest that both purine and thiamine biosynthesis are critical functions of PurF because *in vitro* growth of the *purF*::Tn mutant was only fully restored with exogenous thiamine and hypoxanthine. Complementation only partially reversed attenuation of the *purF*::Tn mutant in mice, despite full restoration of *in vitro* growth without nutrient supplementation. This suggests that *M*. *tuberculosis* experiences greater demand for purine and/or thiamine during infection and that even partial loss of PurF function limits *M*. *tuberculosis* growth in the host. Indeed, CRISPRi transcriptional knock-down of *purF* caused attenuation of *M*. *tuberculosis* in macrophages [57]. However, it is not clear whether *M*. *tuberculosis* requires *de novo* purine and/or thiamine synthesis only for replication during acute infection or also for persistence during chronic infection. Whether an inability to produce thiamine contributes to the attenuation of the *purF*::Tn mutant also remains to be determined. Our future studies will explore these questions using *M*. *tuberculosis* strains that conditionally express enzymes specific to the purine or thiamine biosynthetic pathways or that are required for both pathways, like PurF.

Our data indicate that loss of either PheA or PurF function is cidal to *M*. *tuberculosis* without nutrient supplementation, but the molecular mechanisms leading to death remain to be determined. For other *M*. *tuberculosis* auxotrophs, loss of viability was correlated with multiple effects on microbial physiology [18,19]. Reduced Phe production in the *pheA*::Tn mutant may deplete chorismate because chorismate mutase is normally feedback inhibited by Phe [71]. Chorismate is a precursor for synthesis of Trp, menaquinones and the mycobactin siderophore, so its depletion would affect multiple processes [48]. Loss of PurF function may be cidal in the absence of exogenous nutrients due to reduced production of both purine nucleotides, which are needed for DNA synthesis, RNA synthesis and energy storage [61], and thiamine, a co-factor for multiple enzymes in central carbon metabolism and branched chain amino acid biosynthesis [69]. Alternatively, the *purF*::Tn mutant may be more susceptible to exogenous stress, including oxygen limitation, as reported for a *Mycobacterium smegmatis purF* mutant [72]. The mechanisms by which starvation for these nutrients cause death of *M*. *tuberculosis* will be explored in our future studies.

In addition to the *purF*::Tn and *pheA*::Tn mutants, our Tn-seq screen identified several other Tn mutants with strong fitness defects in mice. These included Tn insertions in genes required for synthesis of riboflavin (*ribA2*, *ribG*), PABA (*pabB*), and the amino acids Trp, Pro and Glu (*trpB*, *trpG*, *proC*, *gltB*), suggesting that *M*. *tuberculosis* cannot acquire these nutrients from host tissues. Our data are consistent with prior studies which showed that *M*. *tuberculosis* requires Trp and Pro biosynthesis during infection [13,20]. Our data also correlate with attenuation of *M*. *tuberculosis* in macrophages upon CRISPRi knock-down of *ribA2*, *trpB*, or *gltB* transcription [57] and with evidence that *M*. *tuberculosis* cannot meet its metabolic demand for Pro by uptake from macrophages [56].

Our data suggest that *M*. *tuberculosis* requires riboflavin, Glu and PABA synthesis for acute infection and that these pathways should be considered as targets for development of new anti-tubercular drugs. An *in vitro* CRISPRi screen identified *ribA2* and *gltB* among the genes most vulnerable to inhibition [9], suggesting that compounds targeting RibA2 or GltB could be effective antibiotics. The riboflavin, Glu and PABA synthesis pathways are also absent from mammals, so drugs targeting these pathways are expected to have low toxicity, and a *M*. *tuberculosis* PabB inhibitor has been reported [73]. Future studies will address whether loss of these metabolic functions is bactericidal and whether *M*. *tuberculosis* also requires these pathways during chronic infection using conditional expression strains.

*M*. *tuberculosis* requires most of the M-ES genes that we analyzed for fitness in mice except *ilvA*. IlvA catalyzes the first step in synthesis of Ile, a branched-chain amino acid (BCAA) [53]. BCAA biosynthesis has been pursued as an antibiotic development target because these pathways are absent in mammals [52]. *M*. *tuberculosis ilvA* is highly vulnerable to inhibition *in vitro* [9], but our data suggest that IlvA would be a poor drug target because the *ilvA*::Tn mutant replicated and disseminated in aerosol-infected mice. Our data are consistent with the observation that a *M*. *tuberculosis* mutant lacking *ilvB1*, which is required for synthesis of all BCAAs (Ile, Leu, Val), was not cleared from intravenously-infected mice [74]. Our data suggest that *M*. *tuberculosis* can either synthesize Ile by an alternate route or obtain sufficient Ile from host tissues. Some bacteria synthesize Ile via a citramalate intermediate [54], but *M*. *tuberculosis* does not encode a citramalate synthase and its LeuA enzyme does not efficiently catalyze this reaction [75]. Isotopic labeling and metabolic flux analysis indicated that *M*. *tuberculosis* can acquire all BCAAs, including Ile, from infected macrophages [56], suggesting that *M*. *tuberculosis* may overcome loss of IlvA function by scavenging Ile from the host. Our data highlight the importance of testing whether biosynthetic pathways essential for *in vitro* growth are also required during infection prior to pursuing these pathways for drug development.

Finally, our work highlights a new strategy to identify genes *M*. *tuberculosis* requires during lung infection using defined Tn mutant pools and quantification of Tn mutant fitness with deep sequencing. Most genome-wide screens of *M*. *tuberculosis* Tn mutants in the mouse model used complex Tn mutant libraries, which enabled analysis of Tn mutant fitness only in the spleen due to a strict lung colonization bottleneck [12–14,16]. Initial Tn mutant screens conducted by signature-tagged mutagenesis used low-complexity Tn mutant pools to identify *M*. *tuberculosis* Tn mutants attenuated in the lungs [17,76,77]. Several recent studies have examined fitness of *M*. *tuberculosis* deletion mutants in the lungs using sequencing barcodes [78,79]. However, to our knowledge this is the first study to use low-complexity Tn mutant pools and Tn-seq to identify *M*. *tuberculosis* mutants attenuated in mouse lungs. We used IV injection to enable analysis of Tn mutant fitness in lungs and spleens, which likely initiates infection in distinct cell types as compared to the aerosol route, in which *M*. *tuberculosis* infects alveolar macrophages. We expect a similar Tn-seq strategy using low-complexity Tn mutant pools and high-dose aerosol infection could be used to identify *M*. *tuberculosis* genes that are required for aerosol transmission or for fitness within lung tissues.

Overall, our findings directly demonstrate that *M*. *tuberculosis* requires both Phe synthesis and *de novo* purine/thiamine synthesis for growth in infected mice, implicating these metabolic pathways as prime targets for development of new TB drugs. In contrast, our data show that *M*. *tuberculosis* does not require IlvA to replicate in aerosol-infected mice, suggesting that Ile biosynthesis should be de-prioritized as a drug development target. The results of our screen also indicate that *M*. *tuberculosis* requires riboflavin, Glu and PABA biosynthesis for growth in host tissues, suggesting further characterization of these metabolic pathways is warranted. Overall, our results widen the range of potential targets for TB drug development.

## Materials and methods

### Ethics statement

All animal protocols used in this study were reviewed and approved by the University of Minnesota Institutional Animal Care and Use Committee (IACUC) under protocol numbers 1912-37660A and 2210-40465A. The University of Minnesota’s NIH Animal Welfare Assurance Number is D16-00288 (A3456-01), expiration date 04/30/2024. All animal experiments were done in strict accordance with the recommendations in the Guide for the Care and Use of Laboratory Animals of the National Institutes of Health [80].

### Bacterial strains and growth conditions

*M*. *tuberculosis* Erdman wild-type and derivative strains were grown aerobically at 37°C in Middlebrook 7H9 (Difco) liquid medium supplemented with 10% albumin-dextrose-saline (ADS), 0.5% glycerol, and 0.1% Tween-80 or on Middlebrook 7H10 (Difco) agar supplemented with 10% oleic acid-albumin-dextrose-catalase (OADC; BD Biosciences) and 0.5% glycerol unless otherwise noted. Frozen stocks of *M*. *tuberculosis* strains were made by growing cultures to late-exponential phase, adding glycerol to 15% final concentration, and storing at -80°C. Liquid cultures of M-ES Tn mutants or the M-ES Tn library were grown in MtbYM rich medium (MtbYM) pH 6.6 [27] supplemented with 10% OADC and 0.05% tyloxapol or on MtbYM agar plates [27], unless otherwise noted. Antimicrobials were used at the following concentrations: kanamycin (Kan) 25 μg/ml for agar or 15 μg/ml for liquid, hygromycin (Hyg) 50 μg/ml, cycloheximide 100 μg/ml.

### Recovery of Tn mutants from the arrayed Tn mutant library

Tn mutants were recovered from the mapped location in our arrayed Tn mutant library [28] by streaking on MtbYM agar containing Kan and incubating at 37°C for at least three weeks. Up to four individual colonies were picked and grown in 10 ml of MtbYM broth containing Kan at 37°C with aeration. The Tn insertion site was confirmed by PCR using a gene-specific primer 5’ or 3’ of the TA site and a primer specific to the Tn (**S4 Table**) followed by Sanger sequencing of the PCR product. M-ES Tn mutants were screened for secondary point mutations that disrupt PDIM production known to be present in our Tn library by PCR and Sanger sequencing. We screened for mutations in *ppsD* and *ppsE* previously identified in other Tn mutants [28] and for additional common mutations in *ppsB*, *ppsC* and *ppsE* identified by whole-genome sequencing of M-ES Tn mutants (**S4 Table**).

### Whole genome sequencing

The whole genome of each M-ES Tn mutant included in the M-ES library was sequenced by short-read IIlumina sequencing to identify mutations in the phthiocerol dimycocerosate (PDIM) locus that could result in PDIM deficiency. Tn mutants were grown to an OD_600_ of 1.0 in MtbYM broth; genomic DNA (gDNA) was extracted by the CTAB-lysozyme method [81] and cleaned with the Genomic DNA concentrator and cleanup kit 25 (Zymo). gDNA was submitted to SeqCenter (formerly Microbial Genome Sequencing Center, MiGS) (Pittsburgh, PA) for library preparation and Illumina sequencing (151 bp paired-end output, 400 Mbp, 2.67 M reads per sample). To generate a consensus sequence for each strain, paired reads were mapped to the *M*. *tuberculosis* Erdman reference genome (NC_020559.1) using the “map to reference” function in Geneious 2021 software (Biomatters, Ltd.) as previously described [28]. To identify single nucleotide polymorphisms (SNPs) in Tn mutant genomes, the WT Erdman and Tn mutant consensus sequences were aligned in Geneious using the “Align Whole Genomes” function with the default Mauve Genome parameters, as described [28]. Tn mutant genomes were only examined for SNPs in the PDIM locus (*tesA–lppX*). SNPs identified in genes required for PDIM synthesis were confirmed by PCR amplification and Sanger sequencing (**S4 Table**). Tn mutants with confirmed SNPs in the PDIM locus were excluded from the study. To confirm Tn insertion sites in the Tn mutants, reads were mapped to the *Himar1* Tn sequence in Geneious as described above and sequences adjacent to the Tn were compared to the *M*. *tuberculosis* Erdman reference genome. Mutants with more than one Tn insertion were excluded from the study.

### Creation and screening of the M-ES Tn library by Tn-seq

We created our M-ES Tn mutant library using Tn mutants in genes predicted to be conditionally essential in Middlebrook 7H9 medium but not MtbYM rich medium (M-ES genes) [11,27], with a single Tn insertion and no mutations in the PDIM locus based on whole-genome sequencing (**S1 Table**). For positive controls, we isolated Tn mutants in genes known to be required for *M*. *tuberculosis* virulence (*panC*::Tn and *phoP*::Tn) [29,37,38]. For negative controls, we isolated Tn mutants in genes known to be dispensable during mouse infection (*rpfA*::Tn, *rpfE*::Tn, *tadA*::Tn, *pstB*::Tn, *pstC1*::Tn) [39–41]. Each Tn mutant was grown individually in MtbYM broth until late-logarithmic phase (OD_600_ ~1.0) and mixed in equal abundance to make the M-ES Tn library. Frozen stocks of the M-ES Tn library were made by adding glycerol to 15% final concentration and freezing 1 ml aliquots. The remaining Tn library culture was aliquoted into three 10 ml cultures and pelleted by centrifugation (4100 x*g*, 10 min) for gDNA extraction as a total library (Library) control. For the Tn-seq screen, the Tn library was grown from a frozen stock to mid-exponential phase (OD_600_ = 0.5) in MtbYM broth, washed once with PBS containing 0.05% Tween-80 (PBS-T), and resuspended at OD_600_ = 0.05 in PBS-T. Three 9 ml samples of the diluted M-ES Tn library (~10^7^ CFU) were pelleted by centrifugation (4100 x*g*, 10 min) for gDNA extraction as an input (Liquid) control. The diluted M-ES Tn library was serially diluted and plated on MtbYM agar to enumerate total bacterial CFU and to isolate a plated input (Plate) control.

C57BL/6J mice 7 weeks of age were purchased from Jackson Laboratory, USA. Mice were injected with 200 μl of the diluted M-ES Tn library (~2 x10^5^ CFU) via the lateral tail vein to deliver ~10,000 CFU to the mouse lungs. Groups of mice (*n* = 3) were euthanized by CO_2_ overdose at days 1, 7, 21, and 42 post-infection. Lungs and spleens were collected, homogenized in PBS-T, serially diluted, and plated on MtbYM agar containing Kan and cycloheximide to enumerate total bacterial CFU in the sample and to recover at least 10^4^ CFU per plate for Tn-seq analysis. Plates were incubated at 37°C with 5% CO_2_ until the biomass on the agar was confluent, up to four weeks. Plates with at least 10^4^ CFU for the Plate controls and mouse tissue homogenates were flooded with 2 ml of GTE buffer [81], and gently scraped with a plastic 10 μl loop to loosen the biomass. Bacteria were collected by centrifugation (4100 x*g*, 10 min). Genomic DNA was extracted from all input controls (Library, Liquid, Plate) and from plated mouse lung and spleen homogenate samples by the CTAB-lysozyme method [81], cleaned with the Genomic DNA clean and concentrator kit (Zymo), and submitted to the University of Minnesota Genomics Center (UMGC) for Tn-seq library preparation and Illumina sequencing.

### Transposon sequencing (Tn-seq) and data analysis

Tn-seq was performed as previously described [27]. *M*. *tuberculosis* gDNA was fragmented with a Covaris S220 ultrasonicator and a whole genome library was prepared using the TruSeq Nano library preparation kit (Illumina). Library fragments containing Tn junctions were PCR-amplified from the whole genome library using the Tn-specific primer Mariner_1R_TnSeq_noMm and Illumina p7 primer (**S4 Table**). The amplified products were uniquely indexed to allow sample pooling and multiplexed sequencing. A total of 27 Mariner-enriched Tn-seq libraries were created. All libraries were pooled and sequenced on a NextSeq 2K P1 2x150-bp run (Illumina) with 20% PhiX target spiked in to account for the low diversity of Tn-seq libraries. Approximately 117 M pass filter reads were generated for the lane. All expected barcodes were detected and reads were balanced (Mean reads ≈ 2.5 M) with mean quality scores for all libraries ≥Q30.

Tn-seq analysis was done as previously described [28]. Sequencing reads were filtered to remove reads without the Tn sequence “GGACTTATCAGCCAACCTGT”. The 5’ Illumina adaptor sequences were trimmed using BBDuk (https://sourceforge.net/projects/bbmap/). Each trimmed read was cut to 30 bases and sequences not starting with TA were removed. Remaining reads were mapped to the *M*. *tuberculosis* Erdman genome (NC_020559.1) using HISAT2. Mapped reads were counted at each TA insertion site to generate read count tables for TnseqDiff analysis. TnseqDiff normalized the read counts using the default trimmed mean of M values (TMM) normalization method [82,83], and then determined conditional essentiality for each TA insertion site between experimental conditions. TnseqDiff calculated the fold change and corresponding two-sided *P*-value for each TA insertion site [42]. All *P*-values were adjusted for multiple testing using the Benjamini-Hochberg procedure in TnseqDiff. The cut-off values for statistical significance were set at a fold-change of > log_2_ ±2 and an adjusted *P*-value < 0.025.

### Tn mutant complementation

Complementation vectors pMV-*purF* and pMV-*pheA* were in made in the integrating plasmid pMV306hyg [28] by Gibson assembly. Each gene was PCR-amplified (Phusion; New England Biolabs) along with ~300 bases 5’ of the translation start site to include the native promoter using primers designed with 16–20 bases complementary to the gene of interest and 18–22 bases that overlap the NcoI site in pMV306hyg (**S4 Table**). PCR products were purified (Qiagen PCR purification kit) and assembled with pMV306hyg that was linearized by digestion with NcoI using HiFi DNA Assembly Master Mix (New England Biolabs) according to the manufacturer’s instructions followed by Sanger sequencing of the cloned gene. The *purF*::Tn and *pheA*::Tn mutants were electroporated with the corresponding complementation vector as described [84]. Each Tn mutant was grown in 7H9 with the appropriate supplements added (60 μM thiamine and 150 μM hypoxanthine for *purF*::Tn or 0.5% casamino acids for *pheA*::Tn). Transformants were selected on Middlebrook 7H10 agar containing Kan and Hyg without supplements. The complementing plasmid was confirmed by PCR (**S4 Table**).

### Growth curves

Each M-ES Tn mutant in the M-ES Tn library and WT Erdman were grown individually in MtbYM broth to mid-exponential phase (OD_600_ = 0.4–0.6), pelleted by centrifugation (2850 x*g*, 10 min) and washed three times with PBS-T before diluting to OD_600_ = 0.01 in 7H9. Optical density (OD_600_) was measured every 2–3 days.

To confirm conditional essentiality of *purF*::Tn in 7H9, WT, *purF*::Tn and *purF*::Tn pMV-*purF* were grown to mid-exponential phase (OD_600_ = 0.4–0.6) in 7H9 supplemented with 60 μM thiamine and 150 μM hypoxanthine (7H9 +thiam +hypo), pelleted by centrifugation (2850 x*g*, 10 min) and washed three times with PBS-T before diluting to OD_600_ = 0.01 in 7H9 or 7H9 +thiam +hypo. The OD_600_ was measured every 2–3 days. Surviving bacteria were enumerated by serially diluting in PBS-T and plating on Middlebrook 7H10 agar with 10% OADC and 0.5% glycerol. 7H10 agar was additionally supplemented with 60 μM thiamine and 150 μM hypoxanthine for recovery of the *purF*::Tn mutant. At later time points, bacteria in 1 ml of the *purF*::Tn 7H9 culture were collected by centrifugation (3000 x*g*, 10 min), resuspended in 100 μl of PBS-T and plated. Plates were incubated for at least 4 weeks at 37°C before enumerating CFU.

To confirm conditional essentiality of *pheA*::Tn or *ilvA*::Tn in 7H9, WT, *pheA*::Tn, *pheA*::Tn pMV-*pheA*, and *ilvA*::Tn were grown to mid-exponential phase (OD_600_ = 0.4–0.6) in 7H9 supplemented with 0.5% casamino acids (7H9 +cas), pelleted by centrifugation (2850 x*g*, 10 min) and washed three times with PBS-T before diluting to OD_600_ = 0.01 in 7H9, 7H9 +cas, 7H9 + 0.6 mM (100 μg/ml) Phe or 7H9 + 0.76 mM (100 μg/ml) Ile. The OD_600_ was measured every 2–3 days. Surviving bacteria were enumerated by serially diluting in PBS-T and plating on Middlebrook 7H10 agar with 10% OADC and 0.5% glycerol. 7H10 agar was additionally supplemented with 0.5% casamino acids for recovery of the *pheA*::Tn or *ilvA*::Tn mutants. Plates were incubated for at least 4 weeks at 37°C before enumerating CFU.

### Phthiocerol dimycocerosate (PDIM) labeling and detection

PDIM production was measured by a radiolabeling and thin layer chromatography (TLC) method [85]. Cultures grown to mid-logarithmic phase in 10 ml of 7H9 broth with appropriate supplements (7H9 +thiam +hypo for *purF*::Tn, 7H9 +cas for *pheA*::Tn or *ilvA*::Tn) were labeled for 48 hr with 10 μCi of [1-^14^C] propionic acid, sodium salt (American Radiolabeled Chemicals, Inc; specific activity 50–60 mCi/mmol) prior to apolar lipid extraction and detection of the PDIM lipid by TLC as described [28].

### Mouse aerosol infection

For retesting individual Tn mutant and complemented strains, male and female C57BL/6J mice 6–8 weeks of age were purchased from Jackson Laboratory, USA, and infected via the aerosol route using an Inhalation Exposure System (GlasCol) as described [86]. The nebulizer was loaded with a bacterial suspension in PBS-T at OD_600_ = 0.007 for WT and complemented strains or OD_600_ = 0.01 for the *purF*::Tn, *pheA*::Tn, or *ilvA*::Tn mutant strains to deliver ~100 CFU to the lungs. Groups of mice (*n* = 6, 3 female and 3 male) were euthanized by CO_2_ overdose at 1, 7, 21, and 42 days post-infection. Bacterial CFU were enumerated by plating serially diluted lung and spleen homogenates on 7H10 agar with cycloheximide. The 7H10 agar was supplemented with 60 μM thiamine and 150 μM hypoxanthine for recovery of the *purF*::Tn mutant or with 0.5% casamino acids for recovery of the *pheA*::Tn or *ilvA*::Tn mutants. Colonies were counted after at least 4 weeks of incubation at 37°C.

### Statistical analysis

A student’s unpaired t-test (two-tailed) was used for pairwise comparisons between WT, mutant and complemented strains. *P* values were calculated using GraphPad Prism 8.0 (GraphPad Software, Inc). *P* values < 0.05 were considered statistically significant.

## Supporting information

S1 Table *M*. *tuberculosis* Tn mutants in M-ES genes recovered from an arrayed Tn mutant library. (XLSX)

S2 Table Tn-seq read counts at each Tn insertion site for all experimental conditions. (XLSX)

S3 Table Complete TnseqDiff analysis to identify Tn mutants with differential fitness across experimental conditions. Comparisons between conditions are indicated in each tab. Bolded text indicates Tn mutants that met statistical significance cutoffs (log_2_ fold change > ± 2, adjusted *P* value <0.025.
(XLSX)

S4 Table Oligonucleotide primers. (XLSX)

S1 Fig *M*. *tuberculosis* Tn mutants in non-essential regions of M-ES genes or in misannotated genes grow normally in Middlebrook 7H9 medium. Strains grown in MtbYM rich medium were washed twice in PBS-T, then diluted to OD_600_ = 0.01 in Middlebrook 7H9 medium. Growth was monitored by measuring the OD_600_. **(A)** Tn insertions within non-essential gene regions (*pstP*, *fhaA*, *ftsQ*, *ERDMAN_3321*) or **(B)** misannotated genes (*ERDMAN_2739*, *ERDMAN_4254*). Data represent the mean ± standard error of three biological replicates.
(TIF)

S2 Fig TnseqDiff analyses to identify Tn mutants with fitness defects in *in vitro* input controls and mouse spleens. Volcano plots of TnseqDiff statistical analyses of Tn-seq data to determine relative Tn mutant fitness in (**A)** infection input liquid culture (Liquid) vs. M-ES Tn library control (Library), **(B)** plate recovery of infection culture (Plate) vs. M-ES Tn library control (Library), **(C)** spleens at day 1 post-infection (D1 Spleen) vs. plated input control (Plate)**, (D)** spleens at day 7 vs. day 1 post-infection, and **(E)** spleens at day 42 vs. day 1 post-infection. Dashed lines indicate cutoffs for statistical significance of ± 2 log_2_ fold change and adjusted *P* value of <0.025. Tn mutants meeting these significance cutoffs are colored and labeled.
(TIF)

S3 Fig The *M*. *tuberculosis purF*::Tn mutant requires exogenous hypoxanthine and thiamine for *in vitro* growth. Strains were grown in 7H9 supplemented with 60 μM thiamine and 150 μM hypoxanthine, washed with PBS-T, and diluted to OD_600_ = 0.05 in 7H9 broth (wild-type and *purF*::Tn), 7H9 with 150 μM hypoxanthine (*purF*::Tn +hypoxanthine), or 7H9 with 60 μM thiamine and 150 μM hypoxanthine (*purF*::Tn +hypoxanthine +thiamine). Growth was measured by optical density at 600 nm. Data represent means ± standard errors of biological triplicate cultures.
(TIF)

S4 Fig *M*. *tuberculosis* strains tested individually in mice are PDIM-proficient. Thin-layer chromatographic analysis of apolar lipids extracted from cultures of WT Erdman (1) *purF*::Tn (2), *pheA*::Tn (3), *purF*::Tn pMV-*purF* (4), *pheA*::Tn pMV-*pheA* (5), and *ilvA*::Tn (6) labelled with [^14^C]-propionate, which is preferentially incorporated into PDIM.
(TIF)

S5 Fig *M*. *tuberculosis pheA*::Tn and *ilvA*::Tn mutants are Phe and Ile auxotrophs, respectively. Strains were grown in 7H9 supplemented with 0.5% casamino acids, washed with PBS-T, and diluted to OD_600_ = 0.01 in 7H9, 7H9 with 0.5% casamino acids, 7H9 with 0.6 mM Phe or 7H9 with 0.76 mM Ile. Growth was measured by optical density at 600 nm. **(A)** *pheA*::Tn mutant; **(B)** *ilvA*::Tn mutant. Data represent means ± standard errors of biological triplicate cultures.
(TIF)

S1 Data Supporting data files for all graphical figures. This supporting data file includes the individual numerical values used to generate the graphs in Figs 1, 2B, 2C, 3, 4, 5, S1, S3, and S5 and the results of statistical analyses on these data. Data for each figure are provided in separate tabs. Raw data used for generating the volcano plots in Figs 2D–2F and S2 are provided in S3 Table.
(XLSX)

S2 Data Raw images. The raw images used to generate S4 Fig are provided.
(PDF)

S1 File Updated R code for TnseqDiff. (ZIP)
