# Supplementary figures and images for: Transposon sequencing reveals metabolic pathways essential for Mycobacterium tuberculosis infection
Source: PLoS Pathog. 2024 Mar 18;20(3):e1011663. doi: 10.1371/journal.ppat.1011663 (PMC10977890; doi:10.1371/journal.ppat.1011663)

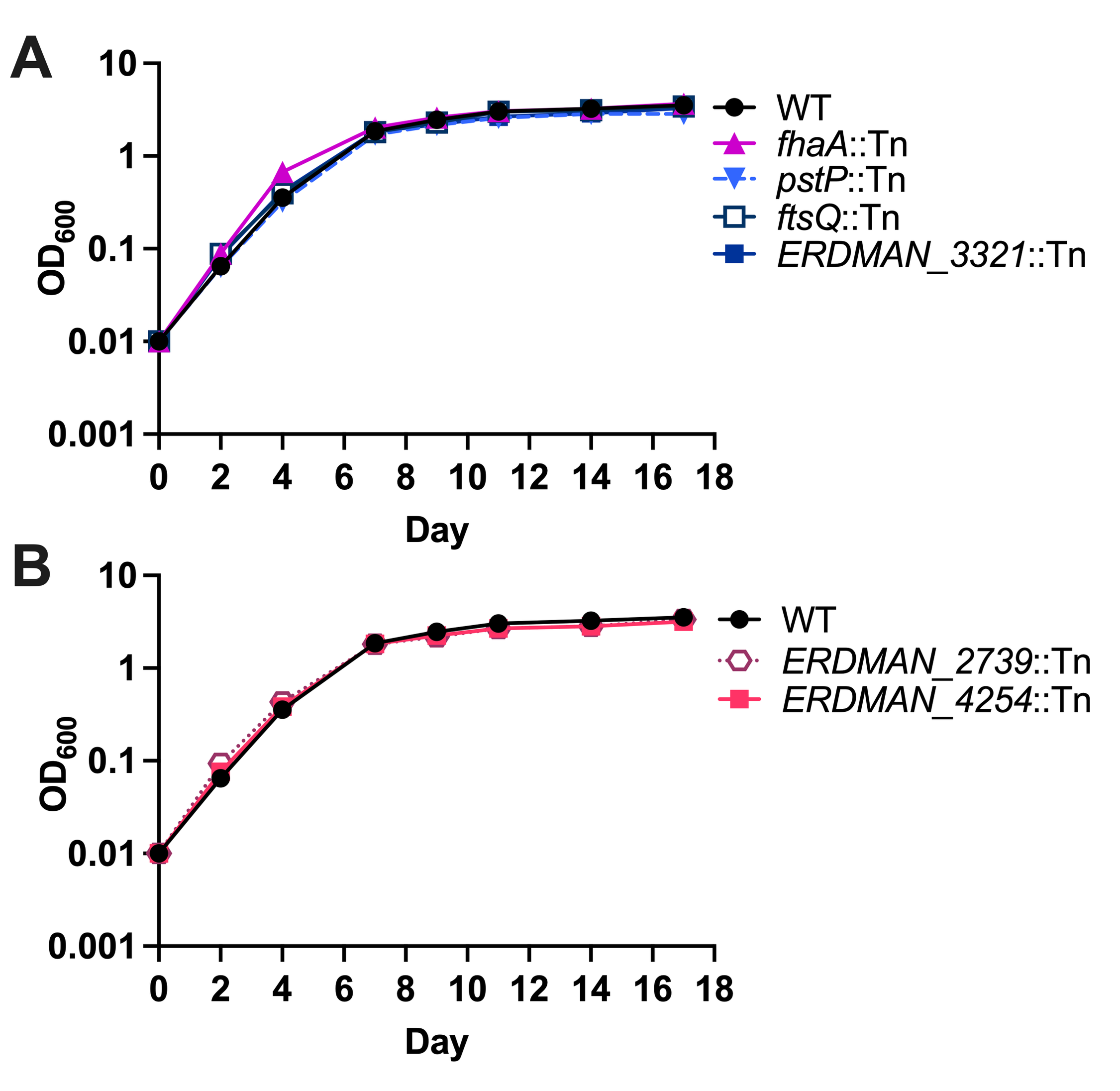

Supplement: S1 Fig — Strains grown in MtbYM rich medium were washed twice in PBS-T, then diluted to OD600 = 0.01 in Middlebrook 7H9 medium. Growth was monitored by measuring the OD600. (A) Tn insertions within non-essential gene regions (pstP, fhaA, ftsQ, ERDMAN_3321) or (B) misannotated genes (ERDMAN_2739, ERDMAN_4254). Data represent the mean ± standard error of three biological replicates. (TIF) [file ppat.1011663.s005.tif]

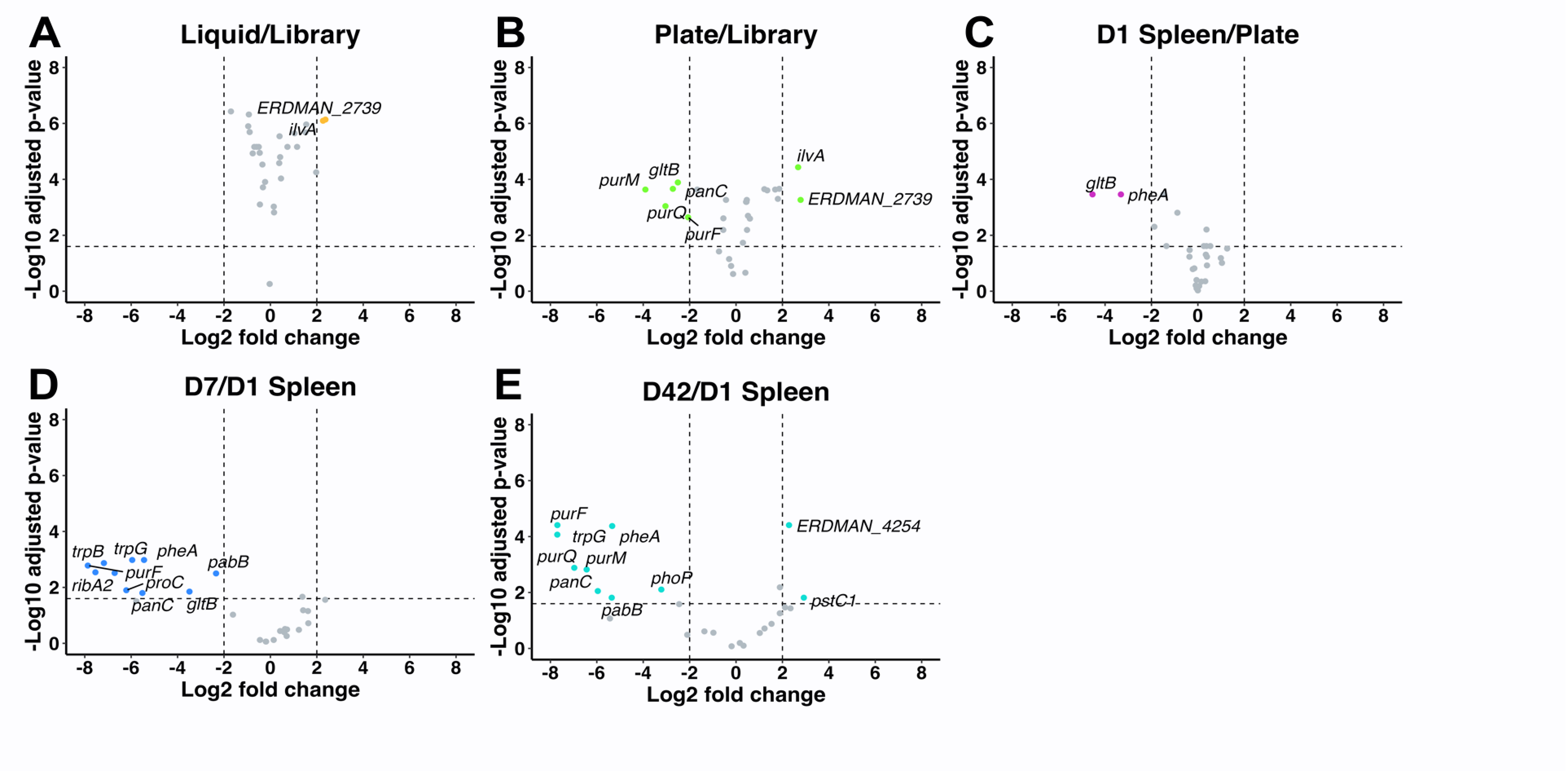

Supplement: S2 Fig — Volcano plots of TnseqDiff statistical analyses of Tn-seq data to determine relative Tn mutant fitness in (A) infection input liquid culture (Liquid) vs. M-ES Tn library control (Library), (B) plate recovery of infection culture (Plate) vs. M-ES Tn library control (Library), (C) spleens at day 1 post-infection (D1 Spleen) vs. plated input control (Plate), (D) spleens at day 7 vs. day 1 post-infection, and (E) spleens at day 42 vs. day 1 post-infection. Dashed lines indicate cutoffs for statistical significance of ± 2 log2 fold change and adjusted P value of <0.025. Tn mutants meeting these significance cutoffs are colored and labeled. (TIF) [file ppat.1011663.s006.tif]

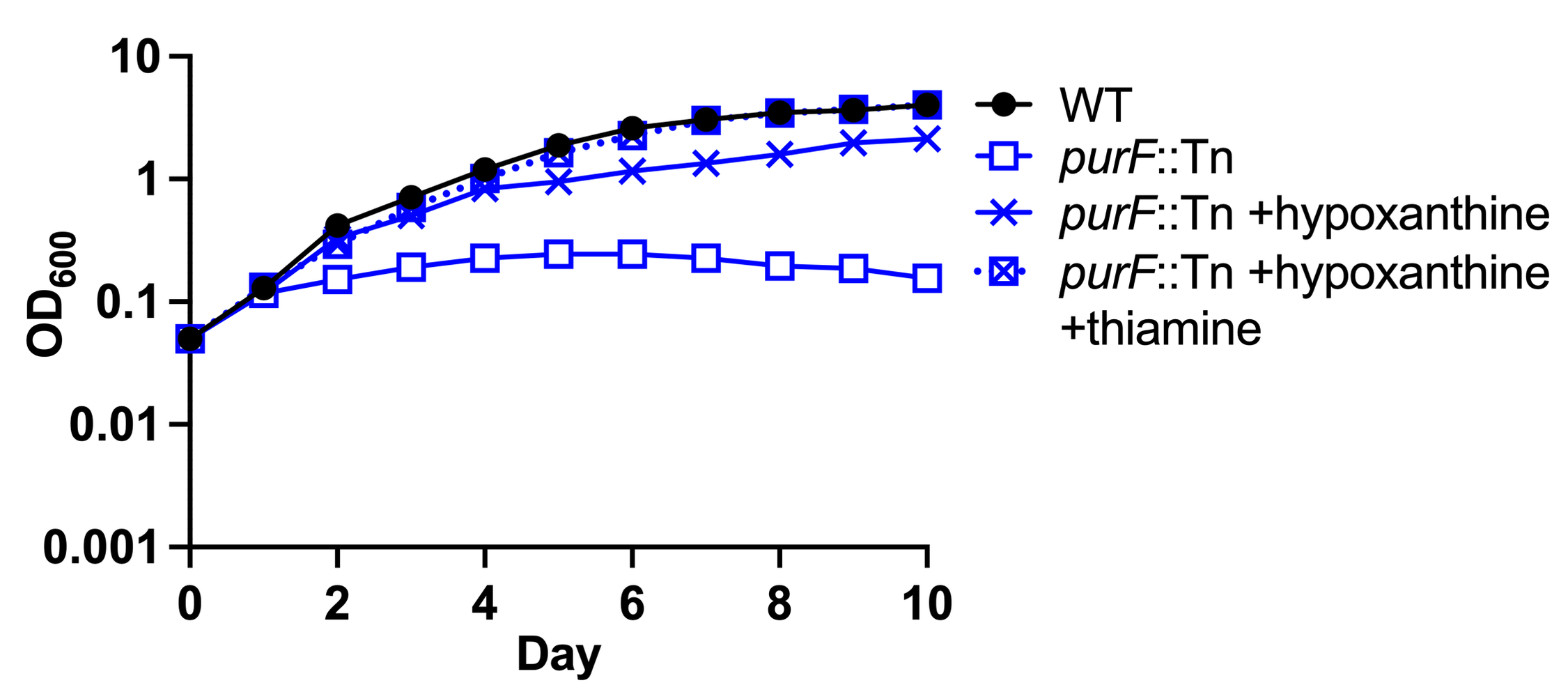

Supplement: S3 Fig — Strains were grown in 7H9 supplemented with 60 μM thiamine and 150 μM hypoxanthine, washed with PBS-T, and diluted to OD600 = 0.05 in 7H9 broth (wild-type and purF::Tn), 7H9 with 150 μM hypoxanthine (purF::Tn +hypoxanthine), or 7H9 with 60 μM thiamine and 150 μM hypoxanthine (purF::Tn +hypoxanthine +thiamine). Growth was measured by optical density at 600 nm. Data represent means ± standard errors of biological triplicate cultures. (TIF) [file ppat.1011663.s007.tif]

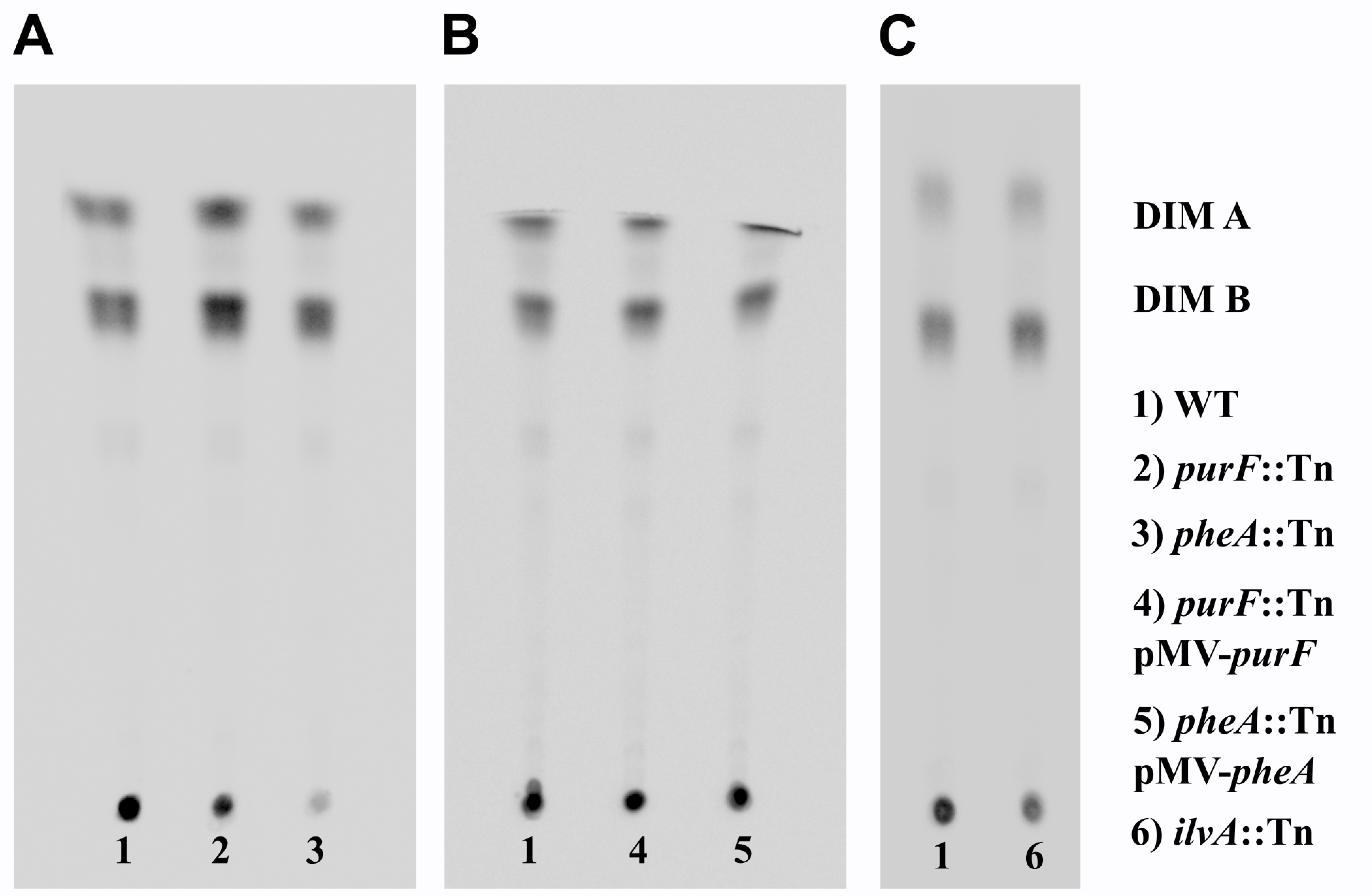

Supplement: S4 Fig — Thin-layer chromatographic analysis of apolar lipids extracted from cultures of WT Erdman (1) purF::Tn (2), pheA::Tn (3), purF::Tn pMV-purF (4), pheA::Tn pMV-pheA (5), and ilvA::Tn (6) labelled with [14C]-propionate, which is preferentially incorporated into PDIM. (TIF) [file ppat.1011663.s008.tif]

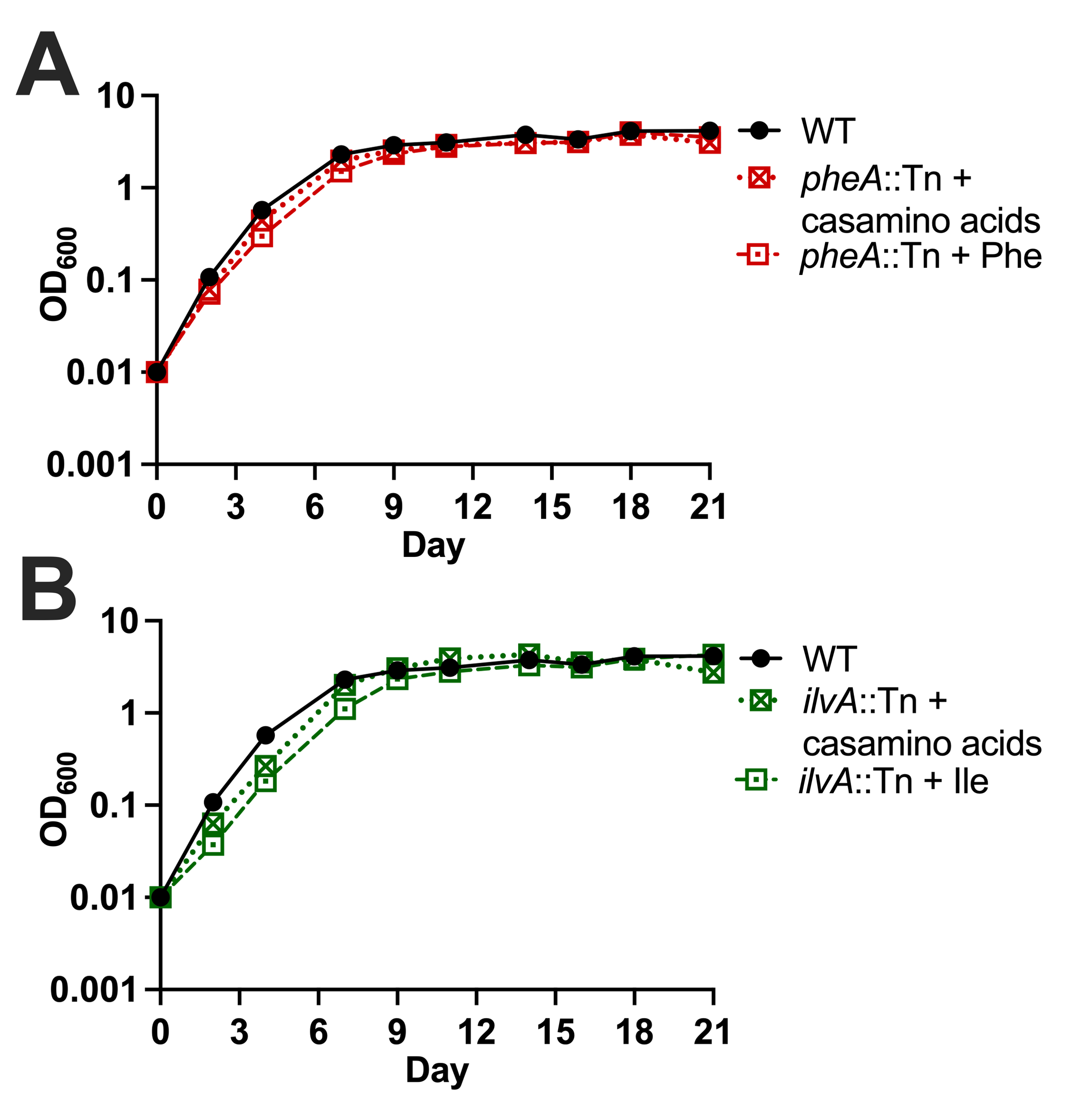

Supplement: S5 Fig — Strains were grown in 7H9 supplemented with 0.5% casamino acids, washed with PBS-T, and diluted to OD600 = 0.01 in 7H9, 7H9 with 0.5% casamino acids, 7H9 with 0.6 mM Phe or 7H9 with 0.76 mM Ile. Growth was measured by optical density at 600 nm. (A) pheA::Tn mutant; (B) ilvA::Tn mutant. Data represent means ± standard errors of biological triplicate cultures. (TIF) [file ppat.1011663.s009.tif]

Fig S4A

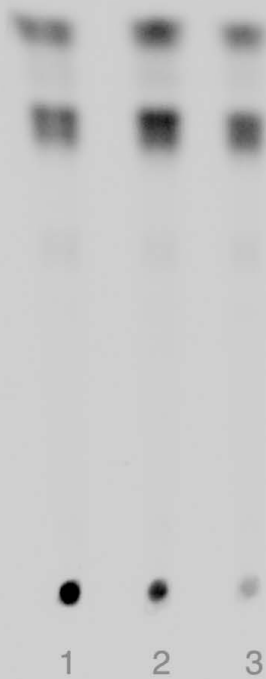

Fig S4B

KODAK STOREAGE PHOSPHOR SCREEN 200530 165400501408W9454

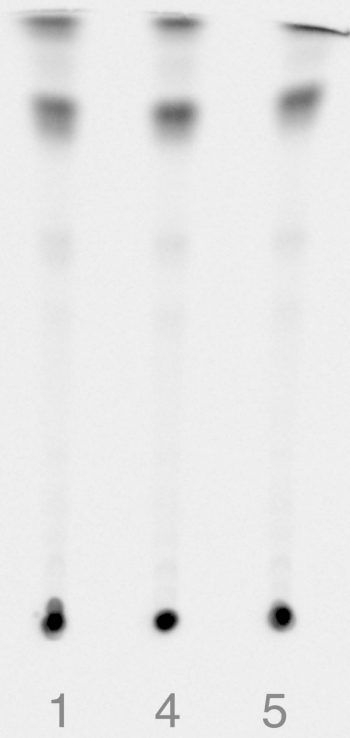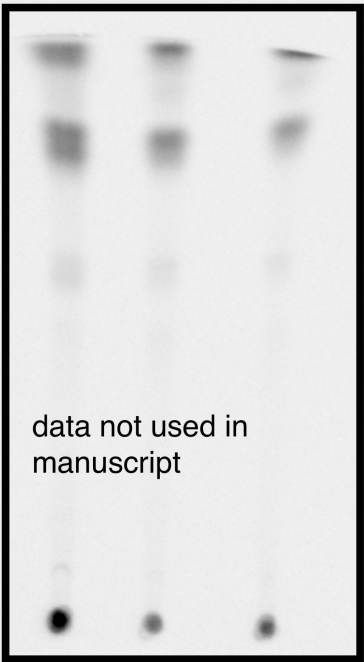

Fig S4C

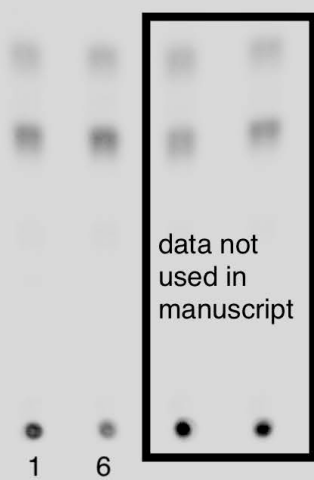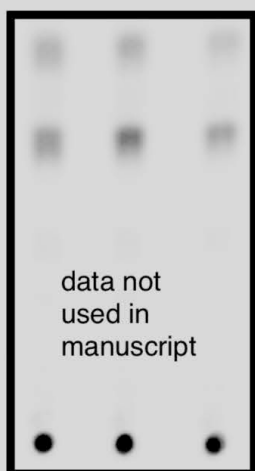

Supplement: S2 Data — The raw images used to generate S4 Fig are provided. (PDF) [file ppat.1011663.s011.pdf]
